# Therapeutic efficacy of sorafenib and plant-derived phytochemicals in human colorectal cancer cells

**DOI:** 10.1186/s12906-023-04032-6

**Published:** 2023-06-26

**Authors:** Abdulmajeed Bahman, Mohamed-Salah Abaza, Sarah Khoushaish, Rajaa J. Al-Attiyah

**Affiliations:** 1 Department of Biological Sciences, Molecular Biology Program, Faculty of Science, Kuwait University, P.O. Box 5969, 13060 Safat, Kuwait; 2 Department of Microbiology and Immunology, Faculty of Medicine, Kuwait University, P.O. Box 24923, 13110 Safat, Kuwait

**Keywords:** Colorectal cancer, Sorafenib, Plant-derived phytochemicals, Combination therapy, Schedule-dependency, Cell cycle / apoptosis-associated proteins

## Abstract

**Background:**

The present study aimed to investigate the sequence-dependent anticancer effects of combined treatment with sorafenib (Sora), a Food and Drug Administration-approved multikinase inhibitor drug, and plant-derived phytochemicals (PPCs) on human colorectal cancer (CRC) cell growth, and proteins associated with the control of cell cycle and apoptosis.

**Methods:**

The cytotoxic effects of 14 PPCs on CRL1554 fibroblast cells were determined using an MTT assay. Moreover, the cytotoxicity of Sora, PPCs, and a combination of both on CRC cells were also investigated. Cell cycle analysis was performed using flow cytometry, and cell apoptosis was investigated using DNA fragmentation, Annexin V/propidium iodide double staining, and mitochondrial membrane potential analyses. The cell cycle- and apoptosis-associated protein expression levels were analysed using western blotting.

**Results:**

Based on their low levels of cytotoxicity in CRL1554 cells at ≤ 20%, curcumin, quercetin, kaempferol, and resveratrol were selected for use in subsequent experiments. The combined treatment of sora and PPCs caused levels of CRC cytotoxicity in a dose-, cell type-, and schedule-dependent manner. Moreover, the combined treatment of CRC cells arrested cell growth at the S and G2/M phases, induced apoptotic cell death, caused extensive mitochondrial membrane damage, and altered the expression of the cell cycle and apoptotic proteins.

**Conclusions:**

Results of the present study highlighted a difference in the level of sora efficacy in CRC cells when combined with PPCs. Further in vivo and clinical studies using the combined treatment of sora and PPCs are required to determine their potential as a novel therapeutic strategy for CRCs.

**Supplementary Information:**

The online version contains supplementary material available at 10.1186/s12906-023-04032-6.

## Background

Colorectal cancer (CRC) is the third most common cancer worldwide and the second leading cause of cancer-related death in the Western world. Most CRC cases are sporadic and may be influenced by environmental factors, including diet. In total, ~ 25% of patients with CRC have a family history of the disease, which may be associated with environmental exposure [1]. Moreover, ~ 35% of patients with CRC exhibit stage IV (metastatic) cancer, and 20–50% of patients with low-stage CRC advance to stage IV, with a ~ 10% five-year survival rate [2]. At present, chemotherapy, surgery, and radiotherapy are used as the main treatment types for CRC [3], and current research focuses on the development of novel treatment options. The limited efficacy of the combination of standard chemotherapy options (FOLFIRI or FOLFOX) with specific monoclonal antibodies (mAbs), such as those against vascular endothelial growth factor (VEGF) or endothelial growth factor receptor, has prompted researchers to seek novel strategies for the treatment of CRC [4].

Research is currently being conducted to identify potential treatment options that target pathways involved in uncontrolled proliferation, neo-angiogenesis, invasion, and metastasis.. Numerous studies have focused on further understanding the kinase activities involved in associated signalling cascades [5]. Moreover, identification of key cancer-associated signalling cascades and protein kinases may result in the development of novel anticancer drugs.

Sorafenib (Sora) is a dual action multikinase inhibitor that targets serine/threonine and receptor tyrosine kinases. It inhibits the Raf signalling cascade, intercepting downstream events that mediate cell growth and proliferation. Sora also interferes with the VEGFR-2 and -3/platelet-derived growth factor receptor β signalling cascades, inhibiting the activation of angiogenesis [6]. Sora inhibits tumor growth and disrupts tumor microvasculature via antiproliferative, anti-angiogenic, and proapoptotic effects [6]. Sora exhibited preclinical and clinical activity against hepatocellular carcinoma (HCC), CRC and renal cell carcinoma [7, 8]. Thus, the specific mechanism of action of Sora highlights its potential as an agent for the therapy of multiple solid tumor types.

Compared with mono-agent therapy, combined therapy that targets multiple signalling cascades may provide an effective alternative to circumvent drug resistance, feedback activation, and compensatory activation of prosurvival cascades [9]. The purpose of this study was to determine whether the PPCs can enhance the chemosensitivity of human colorectal cancer cells to Sora through testing three administration protocols, i.e. sequential, inverted sequential and simultaneous. Furthermore, cell cycle, apoptosis and a panel of proteins associated with their control will be studied.. CRC was selected as a model system for use in the present study as it represents a complex and heterogeneous tumor, displaying several defective signalling cascades [10]. Due to their diverse bioactivities, phenols and polyphenols have been extensively studied and have been used in medicine, prophylaxis and the treatment of other diseases, including several types of cancer. Numerous mechanisms of PPCs leading to the inhibition of cancer growth have been reported [10, 11]. For example, resveratrol (Rsv), phenolic acids, and flavonoids induce apoptosis, whereas Rsv and quercetin (Que) arrest specific phases of the cell cycle in cancer cells. Moreover, curcumin (Cur), Rsv, and epigallocatechin gallate inhibit signalling cascades that induce cellular proliferation.

PPCs provide a promising and pragmatic approach to cancer therapy and may provide a safe and cost-effective alternative to current cancer therapies [12]. Unlike mono-targeted pharmaceutical drugs, PPCs are multitarget agents that modulate cancer growth and progression [13, 14]. PPCs also have a higher margin of safety with negligible cytotoxicity, even at relatively high concentrations [15]. The present study aimed to explore the potential of PPCs, such as Cur, Que, kaempferol (Kmf), and Rsv, in potentiating the anticancer effects of Sora in CRC cells. Moreover, the present study also aimed to identify the most effective PPC and drug combinations, and the associated mechanisms that may potentiate the therapeutic effect of sora on CRC.

## Materials and methods

### Cell lines and chemicals

Human CRC cell lines SW1116 and SW837 and normal human fibroblast BUD8 (CRL1554) were obtained from the American Type Culture Collection. SW1116 and SW837 cells were grown in Leibowitz-15 (L15; 90%; Gibco; Thermo Fisher Scientific, Inc.) containing 10% FBS in a non-CO2 incubator. CRL1554 cells were grown in DMEM (90%; Gibco; Thermo Fisher Scientific, Inc.) containing 10% FBS in a CO2 incubator. Penicillin/streptomycin (Sigma-Aldrich; Merck KGaA), gentamicin (Gibco; Thermo Fisher Scientific, Inc.), L-glutamine (Fluka; Biochemika™), and sodium hydrogen carbonate (BDH; GPR™) were added to all media during preparation. The PPCs were all obtained from Sigma-Aldrich (Merck KGaA), including betulinic acid (BetA), coumarin (Cmr), Cur, hesperetin (Hsp), homoharringtonine (HHG), indol-3-carbinol (IC3), irinotecan (Irt), Kmf, lycopene (Lyp), Que, Rsv, silibinin (Sil), sinigrin (Snn), and sulanidac (Sul). Sora was purchased from MedChemExpress.

### Dose-dependent antiproliferative effects of a panel of PPCs on CRL1554 normal human fibroblasts

The cytotoxicity of a panel of 14 PPCs (Kmf, Cur, Que, Rsv, HHG, Hsp, BetA, IC3, Cmr, Sul, Irt, Lyc, Sil, and Snn) on CRL1554 normal human fibroblast cells was determined using the MTT assay [16]. CRL1554 cells were seeded (27 × 10^3^ cells/well) into 96-well flat-bottomed plates and incubated at 37˚C for 18 h. Subsequently, the medium was removed, and the cells were washed with Hanks balanced salt solution (HBSS; 100 µl/well), and treated with various concentrations of PPCs (20, 40, 60, 80, 100, 120, 140 and 160 µM) for 72 h. The cells were then washed twice with HBSS, and 100 μl medium with 20 µl (5 mg/ml) MTT solution in PBS was added to each well and incubated at 37˚C for 4 h. The supernatants were aspirated, and 200 μL of dimethyl sulfoxide (DMSO) was added to dissolve the formazan crystals. Finally, the absorbance was measured using a multiwell spectrophotometer at two wavelengths (λ, 492 and 570 nm).

### Schedule-dependent cytotoxicity of combined treatments of Sora and PPCs (Cur, Kmf, Que, and Rsv) on SW1116 and SW837 human CRC cell lines

The present study aimed to evaluate the dependency of combined treatment of sora and PPCs on the schedule of administration in CRC cell lines (**17**). For sequential treatment, CRC cell lines SW1116 and SW837 were seeded (27 × 10^3^ cells/well) into 96-well flat-bottomed plates and incubated in a non-CO2 incubator at 37˚C for 18 h. The medium was removed, and the cells were incubated with Sora (0.25–10 µM) at 37˚C for 24 h. The plates were washed, and PPCs [Cur, Kmf, or Que (60 or 120 µM), or Rsv (40 or 80 µM)] were added, followed by incubation at 37˚C for 48 h. For inverted sequential treatment, PPC [Cur, Kmf, or Que (60 or 120 µM), or Rsv (40 or 80 µM)] was added and incubated at 37˚C for 24 h. Subsequently, the plates were washed, and Sora (0.25–10 µM) was added and incubated for 48 h. For simultaneous treatment, Sora (0.25–10 µM) and PPC [Cur, Kmf or Que (60 or 120 µM), or Rsv (40 or 80 µM)] were added simultaneously and incubated in a non-CO2 incubator for 72 h. Cell growth was monitored as previously described.

### Cell cycle analysis of CRC cells treated with Sora, Cur, Kmf, or Que and their simultaneous or sequential combination

Flow cytometry was carried out to monitor the distribution of CRC cells in the different cell cycle phases, following treatment with Sora, Cur, Que or Kmf and their simultaneous or sequential combinations, as previously described [16, 17]. Briefly, CRC cells (2.5 × 10^5^ cells/well) were seeded into 24-well plates in a non-CO2 incubator at 37˚C for 18 h, followed by treatment with single and simultaneous combined treatment with Sora (5 µM) and Cur or Que (200 and 400 µM) for the SW1116 colon cell line, or with sequential combined treatment with Sora (5 µM) followed by Cur or Kmf (200 and 400 µM) for the SW837 rectum cancer cell line. The cells were subsequently processed using a DNA-prep kit and DNA-Prep EPICS workstation. The cells were treated with a non-ionic detergent to permeabilize the cell membrane, followed by propidium iodide (PI) and RNase, and incubated at 15–20˚C for 15 min. The cells were resuspended in binding buffer at a concentration of 3–10 × 10^6^ cells/ml for optimal staining. Fluorescence was measured using a flow cytometer (Beckman Coulter, Inc.), and the percentage of cells in the different phases of the cell cycle was calculated using the Phoenix statistical software package, Advanced DNA cell cycle software (Phoenix Flow System).

### DNA fragmentation assay

Induction of apoptosis was monitored using a DNA fragmentation assay following the manufacturer’s instructions (Apoptotic DNA Ladder Detection kit; Abcam). Briefly, the CRC cell lines SW1116 and SW837 (2.5 × 10^5^ cells/well) were plated into 24-well plates in a non-CO2 incubator at 37˚C for 18 h, followed by a single treatment with Sora (5 µM) and simultaneous treatment with Sora (5 µM) and Cur or Que (200 and 400 µM) for the colon cancer (SW1116), and Cur or Kmf (200 and 400 µM) for the rectum cancer (SW837) cell lines. Cells were subsequently incubated at 37˚C for 72 h. The cells were then trypsinized, harvested, washed, pelleted, and lysed using Tris–EDTA (TE) lysis buffer (35 µl). Enzyme A solution (RNase; 5 µl) was added, followed by incubation at 37˚C for 10 min. Enzyme B solution (proteinase; 5 µl) was then added, followed by incubation at 50˚C for 30 min. Ammonium acetate solution (5 µl) and isopropanol (50 µl) were added to each sample and incubated at -20˚C for 10 min. Finally, the DNA was pelleted, washed with 0.5 ml 70% methanol, and dissolved in 30 µl DNA suspension buffer. The extracted DNA samples were loaded onto a 1.2% agarose gel, along with a loading marker (1 kb DNA ladder), and subjected to electrophoresis, using a running buffer containing 1.35 µg/ml ethidium bromide at 5 V/cm for 1 h. Ethidium bromide-stained DNA bands were visualized using transillumination with UV light.

### Annexin V-fluorescein isothiocyanate (FITC) and PI double staining assay

The levels of cell surface phosphatidylserine were determined to monitor the type of cell death and the kinetics of apoptosis [17]. The CRC cell lines SW1116 and SW837 were seeded (2.5 × 10^5^ cells/well) into 24-well plates and incubated in a non-CO2 incubator at 37˚C for 18 h. Colon cancer (SW1116) cells were simultaneously treated with Sora (5 µM) and Cur or Que (200 and 400 µM) for 72 h, and rectum cancer (SW837) cells were treated with Sora (5 µM) and Cur or Kmf (200 and 400 µM). The cells were subsequently washed twice with HBSS and harvested using trypsin. Finally, the cells were double-stained using the Annexin V-FITC-Flous staining kit, according to the manufacturer’s instructions (Roche Diagnostic GmbH). Annexin V-Flous labelling solution containing Annexin V-FITC and PI (100 µl) was added to both the treated and control cell groups and incubated at 15–20˚C for 15 min. The cells (1 × 10^6^ cells/ml) were then resuspended in binding buffer, and fluorescence was monitored using flow cytometry (FC500; Beckman Coulter, Inc.).

### Mitochondrial membrane potential (MMP) analysis

The inner mitochondrial membrane is rich in negatively charged glycoproteins and is therefore negatively charged. The accumulation of high concentrations of protons outside of the inner membrane establishes the transmembrane potential. The MMP was monitored using the NIR Mitochondrial Membrane Potential Assay kit (Abcam) according to the manufacturer’s protocol. Briefly, cells (2.5 × 105 cells/well) were seeded into 24-well plates and incubated in a non-CO2 incubator at 37˚C for 18 h. The cells were treated for 72 h either simultaneously with Sora (5 µM) and Cur or Que (200 and 400 µM), for SW1116 cells, or sequentially with Sora (5 µM) and Cur or Kmf (200 and 400 µM) for the SW837 cells. MitoNIR Dye (200X; 5 µl/ml) was added to each sample, followed by incubation at 37˚C for 15–30 min. The cells were subsequently pelleted and resuspended in 1 ml assay buffer. Flow cytometry, in the FL4 channel (λExcitation/λEmission, 635/660 nm), was used to monitor the fluorescence intensity.

### Western blot analysis

Proteins associated with the control of both cell cycle and apoptosis were examined using western blot analysis (17), following single and simultaneous treatment of SW1116 cells with sora (5 µM) and Cur (200 and 400 µM). Total cellular proteins were extracted using the Mammalian Cell & Tissue Extraction kit (BioVision, Inc.) according to the manufacturer’s protocols. Total protein concentration was determined using the protein assay kit II (Bio-Rad Laboratories, Inc.). Protein extracts (60 µg) were mixed with 2X Laemmli sample loading buffer and loaded into gels (CriterionTM TGX Stain-freeTM Precast gel; Bio-Rad Laboratories, Inc.) along with Precision Plus ProteinTM dual color standard prestained marker (Bio-Rad Laboratories, Inc.), and subjected to electrophoresis at 250 V for 25 min. The proteins were transferred onto low-fluorescence PVDF membranes using the Trans Blot® TurboTM system (Bio-Rad Laboratories, Inc.) for 7 min, and the blots were checked using the complementary imaging system and software (Image LabTM; version, 5). The membranes were washed three times for 5 min each with Tris-buffered saline containing 0.05% Tween-20 (TBST), and nonspecific binding sites were blocked by incubation with 5% bovine serum albumin-Tris-buffered saline Tween-20 buffer (BSA/TBST; Bio-Rad Laboratories, Inc.) at 37˚C for 1 h. The membranes were subsequently washed again and incubated at 4˚C overnight with the following primary antibodies: Cyclin A2 rabbit mAb (E399; cat. no. ab32498), cyclin B1 XP® rabbit mAb (D5C10; cat. no. 12231), cyclin D1 rabbit mAb (92G2; cat. no. 2978), p27Kip1 XP® rabbit mAb (D69C12; cat. no. 3686), phosphorylated (p)-retinoblastoma protein (p-Rb) rabbit mAb (Ser780; C84F6; cat. no. 3590), cleaved-caspase-3 rabbit mAb (Asp175; 5A1E; cat. no. 9664), cleaved caspase-9 rabbit mAb (Asp330; D2D4; cat. no. 7237), Bax rabbit mAb (D2E11; cat. no. 5023), Bcl extra-large protein (Bcl-xL) rabbit mAb (54H6; cat. no. 2764) and β -actin rabbit mAb (cat. no. 4967; Cell Signalling Technologies, Inc.) at 1:1,000 dilutions in 5% BSA-TBST. The membranes were washed and incubated with horseradish peroxidase-conjugated rabbit anti-mouse immunoglobulin G as the secondary antibody (Cell Signalling Technologies, Inc.) at a 1:2,000 dilution in 5% BSA-TBST at room temperature for 1 h. The membranes were washed in TBST and stained using Bio-Rad ClarityTM western enhanced chemiluminescence substrate mixture (Bio-Rad Laboratories, Inc.; 6 ml peroxide solution + 6 ml Luminol/enhanced solution) in the dark for 5 min. Protein bands were detected using the ChemiDoc™ MP imaging system and Image LabTM software, version 5 (Bio-Rad Laboratories, Inc.). β-actin is commonly used in western blot as loading controls because it is expressed by all eukaryotic cell types and is unaffected by cellular treatments.

### Statistical analysis

Statistical analyses were performed using SPSS (version 25; IBM Corp.). The statistical significance of differences between the control and treated groups was determined using one-way ANOVA and Fisher’s least-significant difference test. P < 0.05 was considered to indicate a statistically significant difference.

## Results

### Dose-dependent antiproliferative effects of Sora and PPCs on CRL1554 normal human fibroblast cells

**Fig. 1:**
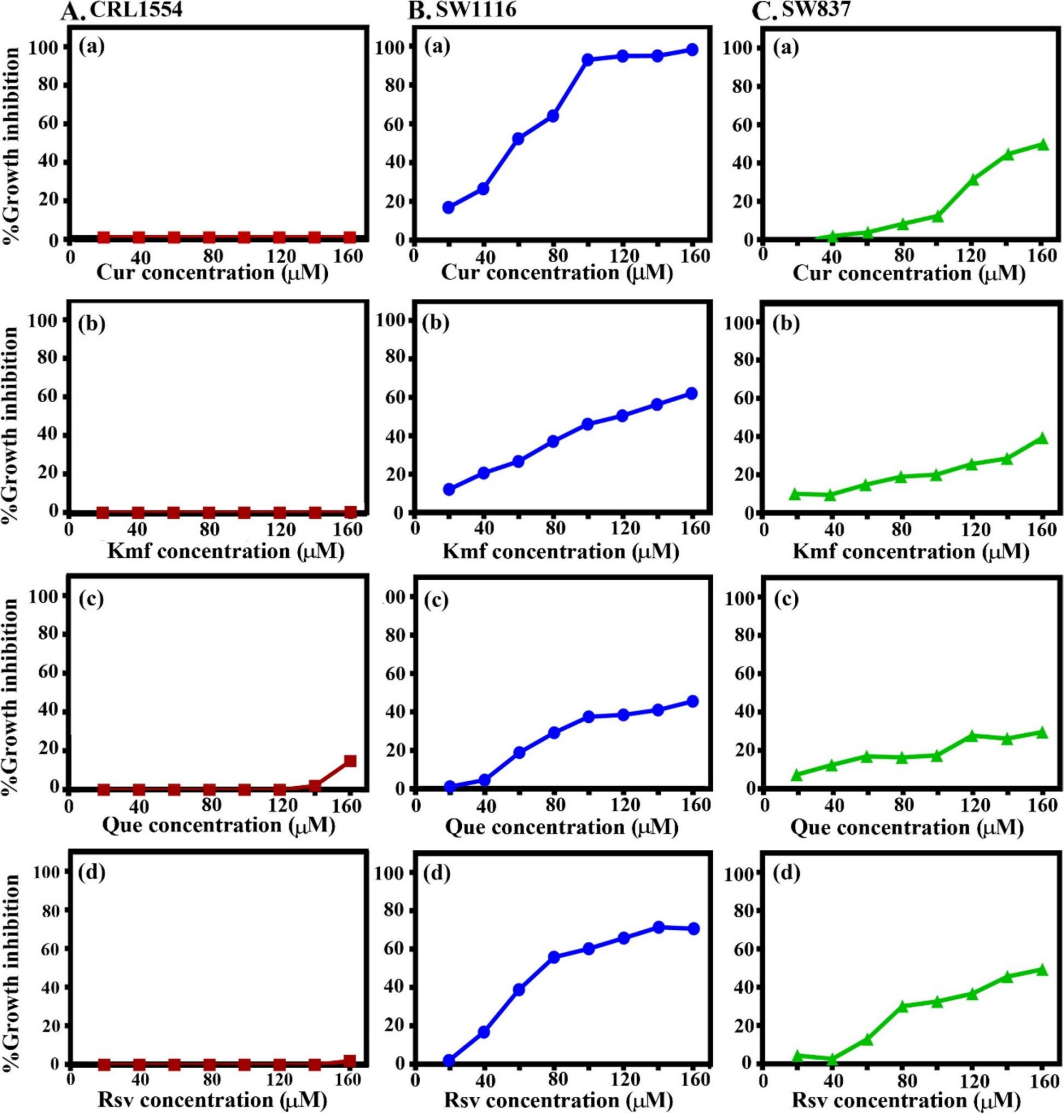
Dose-dependent antiproliferative effect of Cur, Kmf, Que, and Rsv on the (**A**) normal human fibroblast CRL1554, (**B**) human colon cancer SW1116, and (**C**) human rectum cancer SW837 cell lines. Cur, curcumin; Kmf, kaempherol; Que, quercetin; Rsv, resveratrol

To test the potential cytotoxicity of Sora and PPCs, CRL1554 normal human fibroblast cells were treated for 72 h with Sora (0.25–10 µM) and PPCs (20–160 µM), including Kmf, Que, Rsv, Cur, Irt, Sil, Snn, Sul, Lyp, Hsp, BetA, Cmr, I3C, and HHG. Results of the present study demonstrated that PPCs exerted differential antiproliferative effects on CRL1554 cells (Fig. 1).

The PPCs can be classified into two groups: I) Those with no or negligible effects (cytotoxicity, ≤ 20%) on CRL1554 cells, including Cur (0.0%; Fig. 1A-a), Kmf (0.0–1.1%; Fig. 1A-b), Que (0.0–16.2%; Fig. 1A-c) and Rsv (0.0–3.3%, Fig. 1A-d), as well as Hsp (0.0–4.4%), Sin (0.0–5.54%), Snn (0.0–7.3%), I3C (0.2–9.2%) and Cmr (7.4–11.34%; results not shown); and II) those with inhibitory effects on the growth of CRL1554 cells, including Lyp (39.7–66.6%), Irt (44.9–81.9%), Sul (38.94–98%), BetA (69–97.5%) and HHG (99–100%; results not shown). The present study aimed to examine the potential of Cur, Que, Rsv, and Kmf in enhancing the lethality of Sora against human CRC, based on their negligible cytotoxic effects in CRL1554 cells.

### Dose-dependent growth inhibitory effects of Cur, Kmf, Que, and Rsv on CRC cell lines

To examine the cytotoxicity of the selected PPCs (Cur, Kmf, Que, and Rsv) on CRC cell lines, SW1116, and SW837 cells were treated with Cur, Kmf, Que, and Rsv (60 or 120 µM). Results of the present study demonstrated that the aforementioned PPCs inhibited the growth of SW1116 cells as follows: Cur (IC50, 60 µM), Kmf (IC50, 100 µM), Que (IC50, 160 µM), and Rsv (IC50, 70 µM; Fig. 1B). Moreover, the aforementioned PPCs also exhibited reduced anticancer effects in the rectum cancer cell line SW837, compared with the colon cancer cell line SW1116. The anticancer effects of these PPCs on SW837 cells were as follows: Cur (IC50, 150 µM), Rsv (IC50, 140 µM), Kmf (cytotoxicity mean, 21 ± 3.5%), and Que (cytotoxicity mean, 19 ± 3%; Fig. 1C). Time dependency of PPC anticancer effects would be examined in future studies.

### Schedule- and dose-dependent anticancer effects of the combined treatment of Sora with Cur, Kmf, Que, or Rsv on SW1116 and SW837 human CRC cell lines

**Fig. 2:**
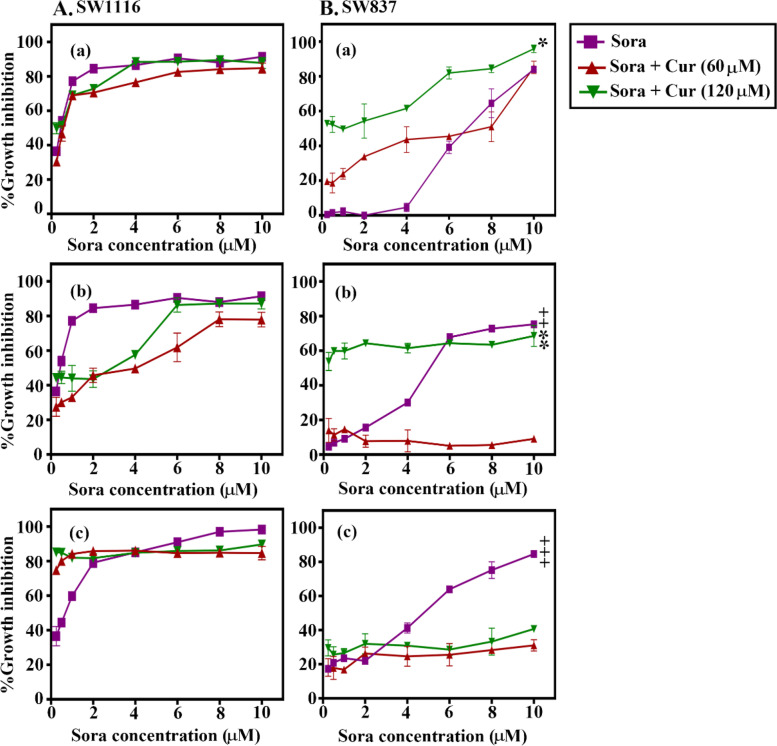
Sequence-dependent antiproliferative effects of the combined treatment of Sora and Cur on (**A**) SW1116 and (**B**) SW837 human colorectal cancer cell lines. Cells were treated with a combination of Sora (0–10 μM) and Cur (60 and 120 μM) in (a) sequential, (b) inverted sequential or (c) simultaneous manners. Cell growth was analysed using the MTT assay. (Aa) SW1116, sequential treatment [Sora + Cur (120 µM)] vs. Sora, *P < 0.05. (Bb) SW837, inverted sequential treatment [Sora + Cur (120 µM)] vs. Sora, **P < 0.05. (Bc) SW837, simultaneous treatment [Sora vs. Sora + Cur (60 µM) or Sora + Cur (120 µM)], ***P < 0.05. Sora, sorafenib; Cur, curcumin

**Fig. 3:**
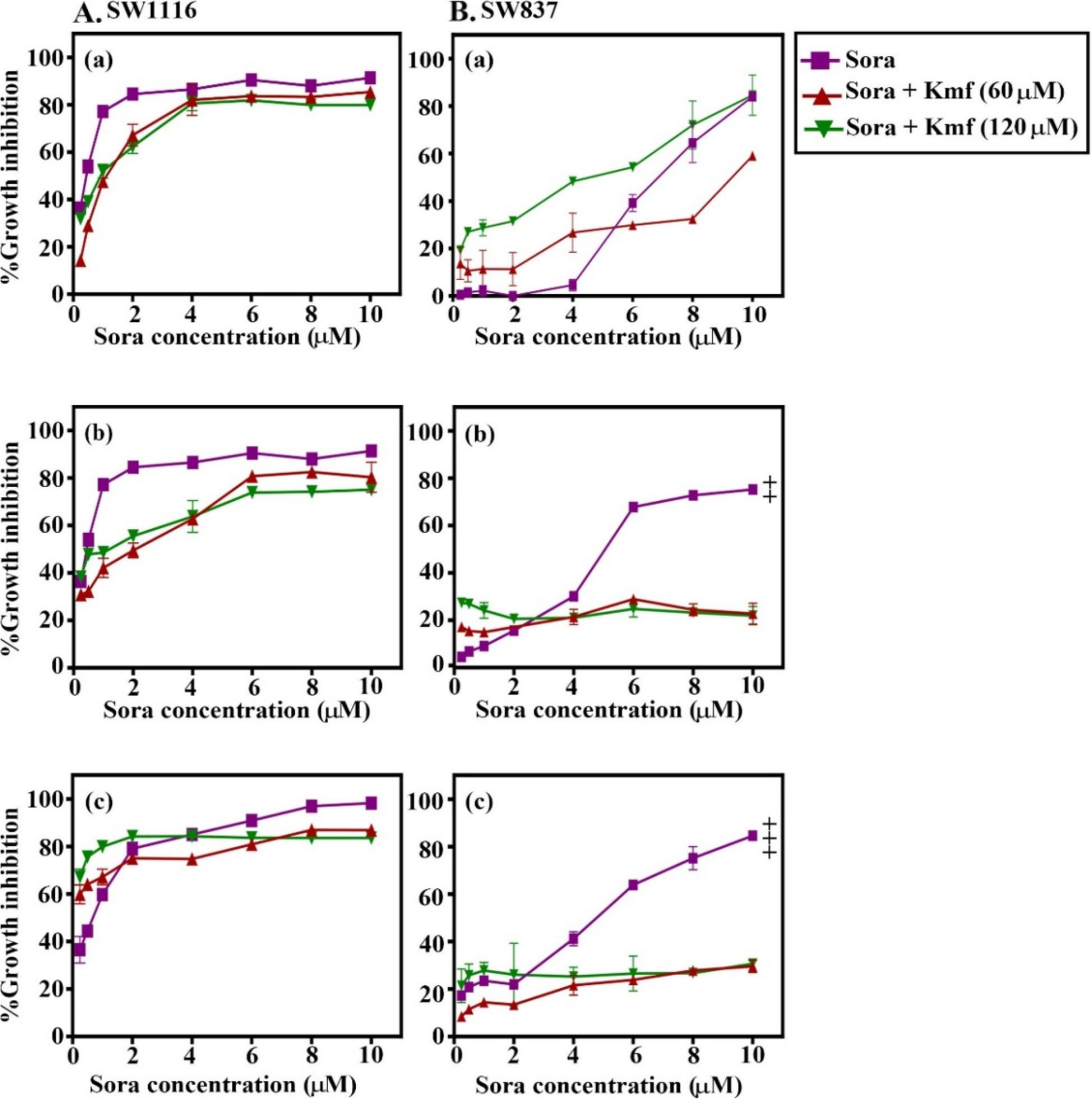
Sequence-dependent antiproliferative effects of the combined treatment of Sora with Kmf on (**A**) SW1116 and (**B**) SW837 human colorectal cancer cell lines. Cell lines were treated with a combination of Sora (0–10 μM) and Kmf (60 and 120 μM) in (a) sequential, (b) inverted sequential, and (c) simultaneous manners. Cell growth was analysed using the MTT assay. (Bb) SW837, inverted sequential treatment (6–10 µM). [Sora + Kmf (60 µM)] vs. Sora or [Sora + Kmf (120 µM)], *P < 0.05. (Bc) SW837, concomitant treatment (6–10 µM). Sora vs. [Sora + Kmf (60 µM)] or [Sora + Kmf (120 µM)], **P < 0.05. Sora, sorafenib, Kmf, kaempferol

**Fig. 4:**
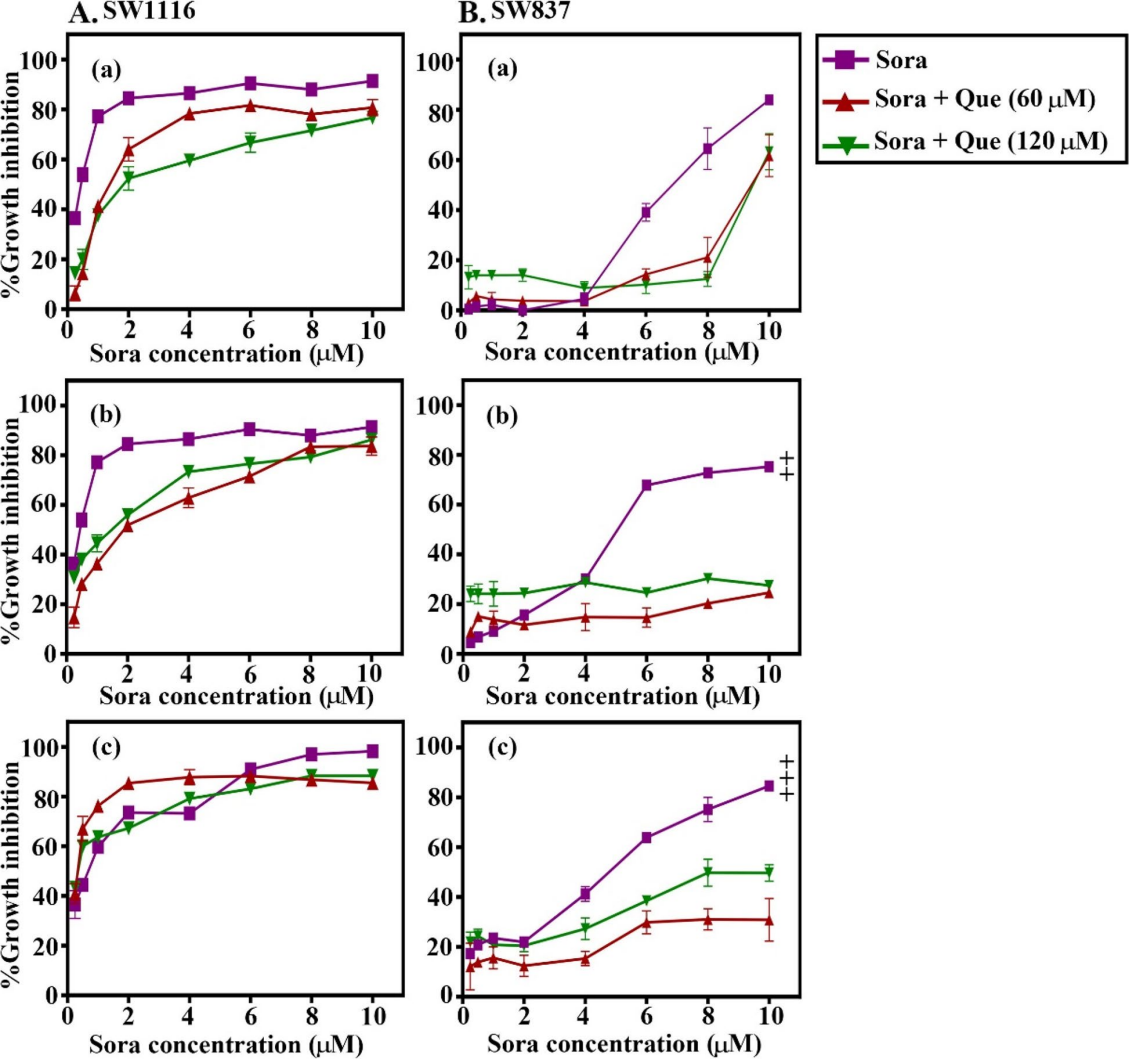
Sequence-dependent antiproliferative effects of the combined treatment of Sora with Que on (**A**) SW1116 and (**B**) SW837 human colorectal cancer cell lines. Cell lines were treated with a combination of Sora (0–10 μM) and Que (60 and 120 μM) in (a) sequential, (b) inverted sequential or (c) simultaneous manners. Cell growth was analysed using the MTT assay. (Bb) SW837, inverted sequential treatment (6–10 µM). Sora vs. [Sora + Que (60 µM)] or [Sora + Que (120 µM)], *P < 0.05. (Bc) SW837, simultaneous treatment (6–10 µM). Sora vs. [Sora + Que (60 µM)], **P < 0.05. Sora, sorafenib; Que, quercetin

**Fig. 5:**
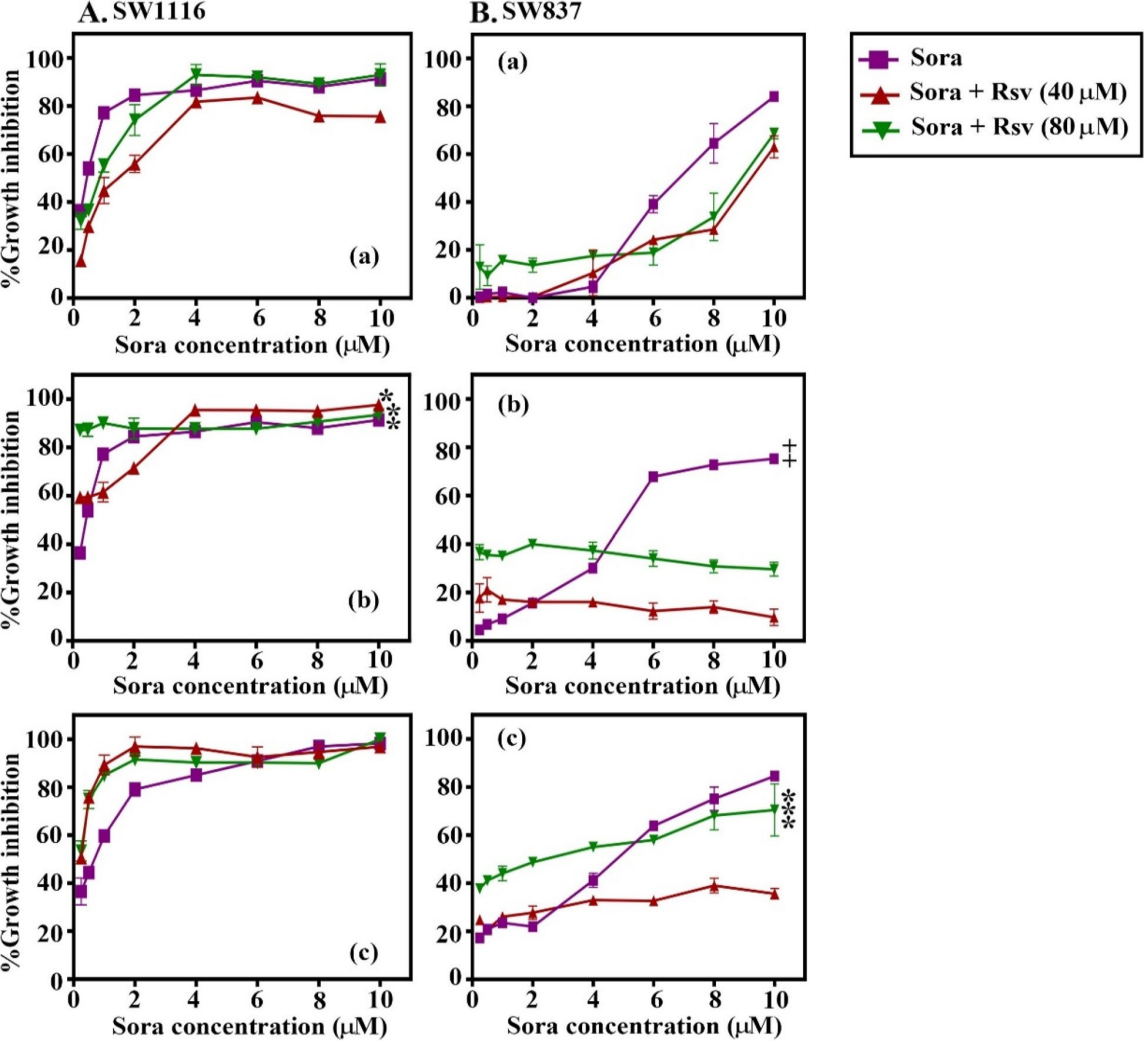
Sequence-dependent antiproliferative effects of the combined treatment of Sora with Rsv on SW1116 (**A**) and SW837 (**B**) human colorectal cancer cell lines. Cell lines were treated with a combination of Sora (0–10 μM) and Rsv (40 and 80 μM) in (a) sequential, (b) inverted sequential or (c) simultaneous manners. Cell growth was analysed using the MTT assay. (Ab) SW1116, inverted sequential treatment [Sora + Rsv (40 µM)] vs. Sora, *P < 0.05. (Ab) SW1116, inverted sequential treatment [Sora + Rsv (80 µM)] vs. Sora, ^**^P < 0.05. (Bb) SW837, inverted sequential treatment (6–10 µM). Sora vs. [Sora + Rsv (40 µM)] or [Sora + Rsv (80 µM)], ***P < 0.05. (Bc) SW837, simultaneous treatment [Sora (0.25–4 µM) + Rsv (40 µM)] or [Sora (0.25 − 4 µM) + Rsv (80 µM)] vs. Sora, + *P* < 0.05. (Bc) Sora (6–10 µM) vs. simultaneous treatment with Sora [Sora (6–10 µM) + Rsv (40 µM)], +  + *P* < 0.05. Sora, sorafenib, Rsv, resveratrol

Sequential, inverted, sequential, and simultaneous combinations of Cur, Kmf, Que, and Rsv were used to examine the anticancer effects of Sora on human CRC cells. The results displayed in Figs. 2, 3, 4 and 5 and Tables 1 and 2 demonstrated that the aforementioned PPCs potentiated the cytotoxicity of Sora in a dose-, CRC cell type-, PPC type- and schedule-dependent manner. Notably, cur and Kmf exhibited comparable effects, which were more pronounced than the effects of que, and these effects were more pronounced than those of Rsv. Based on the results of the cytotoxicity assay, the simultaneous treatment of Sora and Cur or que on the SW116 colon cancer cell line, and the sequential treatment of Sora and Cur or Kmf on the SW837 rectum cancer cell line exhibited the highest cytotoxic effects. These combinations were therefore used in subsequent studies to investigate the potential molecular mechanisms of the combined treatments.

**Table 1:** IC-values, SR, and *P*-values of single and combined treatment with Sora and Cur or Kmf in human colorectal cancer cell lines

Single and combined treatment with Sora and Cur or Kmf	SW1116			SW837		
**A. Single and combined treatment with Sora and Cur.**	**IC-values (µM)**	**SR**	***P*-value**	**IC-values (µM)**	**SR**	***P*-value**
**1. Sequential treatment with Sora and Cur: Sora (24 h) followed by Cur (48 h)**						
a. Sora (0.25- 10 µM)	IC_60_ = 7.70	N.A	-	IC_70_ = 0.86	N.A	-
b. Sora (0.25- 10 µM) + Cur (60 µM)	IC_60_ = 9.14	0.843	0.307	IC_70_ = 0.86	1.00	0.993
c. Sora (0.25- 10 µM) + Cur (120 µM)	IC_60_ = 4.00	1.925	0.006	IC_70_ = 0.57	1.51	0.457
**2. Inverted sequential treatment with Sora and Cur: Cur (24 h) followed by Sora (48 h)**						
a. Sora (0.25- 10 µM)	IC_70_ = 6.00	N.A	-	IC_60_ = 8.00	N.A	0.081 *vs*. b 0.001 *vs*. c
b. Sora (0.25- 10 µM) + Cur (60 µM)	IC_70_ = 6.86	0.880	0.504	N.D	N.D	b
c. Sora (0.25- 10 µM) + Cur (120 µM	IC_70_ = 4.86	1.240	0.504	IC_60_ = 2.00	4.00	c
**3. Simultaneous treatment with Sora and Cur: (72 h)**						
a. Sora (0.25- 10 µM)	IC_80_ = 4.86	N.A	-	IC_60_ = 7.43	N.A	0.019 *vs*. b 0.027 *vs*. c
b. Sora (0.25- 10 µM) + Cur (60 µM)	IC_80_ = 0.57	8.530	0.136	N.D	N.D	b
c. Sora (0.25- 10 µM) + Cur (120 µM	IC_80_ = 0.57	8.530	0.088	N.D	N.D	c
**B. Single and combined treatment with Sora and Kmf**	**IC-values (µM)**	**SR**	***P*- value**	**IC-values (µM)**	**SR**	***P*-value**
**1. Sequential treatment with Sora and Kmf: Sora (24 h) followed by Kmf (48 h)**						
a. Sora (0.25- 10 µM)	IC_60_ = 0.57	N.A	-	IC_60_ = 7.43	N.A	0.716 *vs*. b
b. Sora (0.25- 10 µM) + Kmf (60 µM)	IC_60_ = 1.14	0.500	0.560	IC_60_ = 9.43	0.79	b
c. Sora (0.25- 10 µM) + Kmf (120 µM)	IC_60_ = 1.14	0.500	0.675	IC_60_ = 6.86	1.08	0.214
**2. Inverted sequential treatment with Sora and Kmf: Kmf (24 h) followed by Sora (48 h)**						
a. Sora (0.25- 10 µM)	IC_60_ = 4.86	N.A	-	IC_60_ = 7.43	N.A	0.010 *vs*. b 0.008 *vs*. c
b. Sora (0.25- 10 µM) + Kmf (60 µM)	IC_60_ = 4.00	1.22	0.148	N.D	N.D	b
c. Sora (0.25- 10 µM) + Kmf (120 µM)	IC_60_ = 2.00	2.43	0.134	N.D	N.D	c
**3. simultaneous treatment with Sora and Kmf: (72 h)**						
a. Sora (0.25- 10 µM)	IC_80_ = 4.86	N.A	-	IC_60_ = 7.14	N.A	0.009 *vs*. b 0.013 *vs*. c
b. Sora (0.25- 10 µM) + Kmf (60 µM)	IC_80_ = 0.57	8.53	0.513	N.D	N.D	b
c. Sora (0.25- 10 µM) + Kmf (120 µM)	IC_80_ = 0.57	8.53	0.152	N.D	N.D	c

*SR* sensitization ratio; the ratio between IC-values of Sora and IC-values of Sora plus PPCs, *N.A* not applicable, *N.D* not determined, *PPCs* plant-derived phenolic compounds

**Table 2:** IC-values, SR, and *P*-values of single and combined treatment with Sora and Que or Rsv in human colorectal cancer cell lines

Single and combined treatment with Sora and Que or Rsv	SW1116			SW837		
**A. Single and combined treatment with Sora and Que.**	**IC-values (µM)**	**SR**	***P*-value**	**IC-values (µM)**	**SR**	***P*-value**
**1. Sequential treatment with Sora and Que: Sora (24 h) followed by Que (48 h)**						
a. Sora (0.25- 10 µM)	IC_60_ = 0.57	N.A	0.337 *vs*. b 0.235 *vs*. c	IC_50_ = 6.86	N.A	0.395 *vs*. b 0.682 *vs*. c
b. Sora (0.25- 10 µM) + Que (60 µM)	IC_60_ = 1.14	0.500	b	IC_50_ = 9.43	0.728	b
c. Sora (0.25- 10 µM) + Que (120 µM)	IC_60_ = 2.00	0.300	c	IC_50_ = 9.43	0.728	c
**2. Inverted sequential treatment with Sora and Que: Que (24 h) followed by Sora (48 h)**						
a. Sora (0.25- 10 µM)	IC_60_ = 4.86	N.A	-	IC_50_ = 6.86	N.A	0.006 *vs*. b 0.010 *vs*. c
b. Sora (0.25- 10 µM) + Que (60 µM)	IC_60_ = 3.43	1.420	0.353	N.D	N.D	b
c. Sora (0.25- 10 µM) + Que (120 µM	IC_60_ = 2.00	2.430	0.152	N.D	N.D	c
**3. simultaneous treatment with Sora and Que:(72 h)**						
a. Sora (0.25- 10 µM)	IC_80_ = 4.00	N.A	-	IC_50_ = 6.57	N.A	0.022 *vs*. b 0.189 *vs*. c
b. Sora (0.25- 10 µM) + Que (60 µM)	IC_80_ = 0.57	7.020	0.581	N.D	N.D	b
c. Sora (0.25- 10 µM) + Que (120 µM	IC_80_ = 0.86	4.650	0.882	10.00	0.66	c
**B. Single and combined treatment with Sora and Rsv**	**IC-values (µM)**	**SR**	***P*-value**	**IC-values (µM)**	**SR**	***P*-value**
**1. Sequential treatment with Sora and Rsv: Sora (24 h) followed by Rsv (48 h)**						
a. Sora (0.25- 10 µM)	IC_60_ = 0.57	N.A	0.522 *vs*. b	IC_50_ = 6.86	N.A	0.452 *vs*. b 0.831 *vs*. c
b. Sora (0.25- 10 µM) + Rsv (40 µM)	IC_60_ = 1.14	0.500	b	IC_50_ = 9.71	0.71	b
c. Sora (0.25- 10 µM) + Rsv (80 µM)	IC_60_ = 1.70	0.340	0.883	IC_50_ = 9.14	0.75	c
**2. Inverted sequential treatment with Sora and Rsv: Rsv (24 h) followed by Sora (48 h)**						
a. Sora (0.25- 10 µM)	IC_60_ = 4.86	N.A	-	IC_50_ = 6.57	N.A	0.003 *vs*. b 0.024 *vs*. c
b. Sora (0.25- 10 µM) + Rsv (40 µM)	IC_60_ = 0.86	5.650	0.001	N.D	N.D	b
c. Sora (0.25- 10 µM) + Rsv (80 µM)	N.D	N.D	0.0001	N.D	N.D	c
**3. simultaneous treatment with Sora and Rsv: (72 h)**						
a. Sora (0.25- 10 µM)	IC_60_ = 1.14	N.A	-	IC_60_ = 7.71	N.A	0.044 *vs*. b 0.964 *vs*. c
b. Sora (0.25- 10 µM) + Rsv (40 µM)	IC_60_ = 0.57	2.00	0.238	N.D	N.D	b
c. Sora (0.25- 10 µM) + Rsv (80 µM)	IC_60_ = 0.57	2.00	0.401	IC_60_ = 6.57	1.170	c

*SR* sensitization ratio; the ratio between IC-values of Sora and IC-values of Sora plus PPCs, *N.A* not applicable, *N.D* not determined, *PPCs* plant-derived phenolic compounds

### Cell cycle analysis of the human CRC cell lines SW1116 and SW837 following single and combined treatments with Sora and Cur, Kmf, or Que

**Fig. 6:**
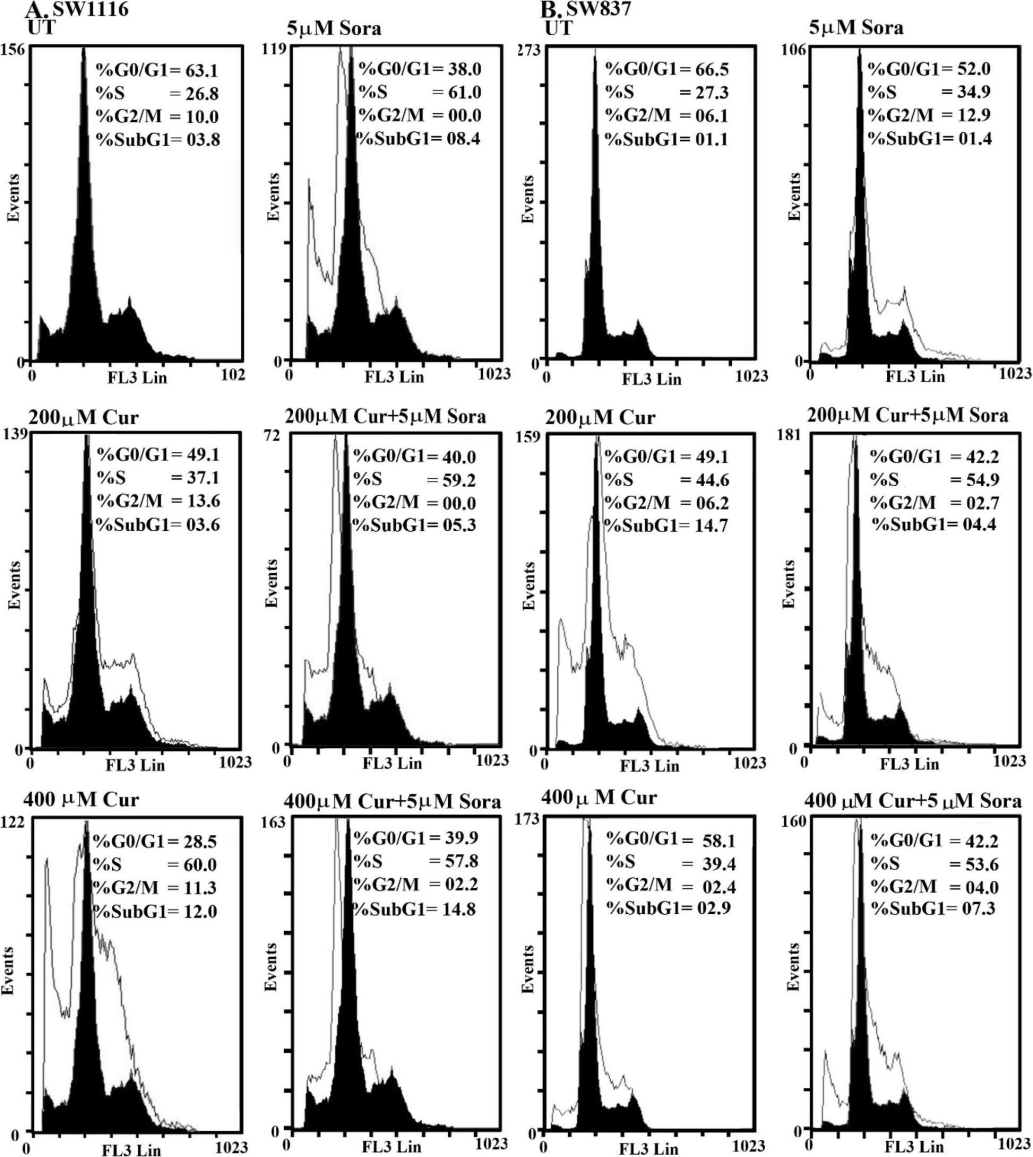
Cell cycle of human CRC cell lines treated with Sora, Cur and a combination of both treatments. **A** The SW1116 human CRC cell line was UT, or treated with Sora (5 µM), Cur (200 µM), Cur (400 µM), and the simultaneous combinations of Sora and Cur (5 + 200 µM) or (5 + 400 µM) for 72 h. **B** The SW837 human CRC cell line was UT, or treated with Sora (5 µM), Cur (200 µM), Cur (400 µM), and the sequential treatments of Sora and Cur (5 + 200 µM) or (5 + 400 µM) for 72 h. At least three samples were analysed, and 20,000 events were scored for each sample. The vertical axis represents the relative number of events, and the horizontal axis represents the fluorescence intensity. The black and white curves are control and experimental groups, respectively. Sora, sorafenib; Cur, curcumin; UT, untreated; CRC, colorectal cancer

The CRC cell line SW1116 was incubated with sora (5 µM), Cur (200 and 400 µM), and their simultaneous combinations for 72 h, and the distribution of cells in the different phases of the cell cycle was determined using flow cytometric analyses. Treatment with Sora (5 µM) markedly arrested the growth of SW1116 cells in the S phase [(61.0 vs. 26.8% for untreated (UT)] with a decrease in the population of cells in both G0/G1 (38.0 vs. 63.1% for UT) and G2/M (0.0 vs. 10.0% for UT). Treatment with Sora also induced apoptotic cell death, as indicated by the percentage of cells in the Sub-G1 phase (8.4 vs. 3.8% for UT; Fig. 6A). Treatment with Cur (200 µM) for 72 h in SW1116 cells arrested cell growth in both the S phase (37.1 vs. 26.8% for UT) and G2/M phase (13.6 vs. 10.0% for UT), accompanied by a decrease in the number of cells in the G0/G1 phases (49.1 vs. 63.1% for UT). The simultaneous treatment of Sora (5 µM) and Cur (200 µM) for 72 h in SW1116 cells markedly arrested the number of cells in the S phase (59.2 vs. 26.8% for UT), while the percentage of cells in both the G0/G1 (40 vs. 63.1% for UT) and G2/M phases were markedly decreased (0.0 vs. 10% for UT). Simultaneous treatment with Sora (5 µM) and Cur (200 µM) also induced apoptosis (cells in sub G1, 5.3 vs. 3.8% for UT; Fig. 6A). Moreover, treatment with Cur (400 µM) markedly arrested the growth of SW1116 cells in the S phase (60 vs. 26.8% for UT); however, cell cycle arrest was only apparent at low levels in the G2/M phase (11.3 vs. 10.0% for UT). There was also a notable decline in the population of cells in the G0/G1 phase (28.5 vs. 63.1% for UT). Treatment with Cur (400 µM) also induced apoptosis in SW1116 cells (cells in sub G1, 12 vs. 3.8% for UT). Moreover, simultaneous treatment with Sora (5 µM) and Cur (400 µM) markedly arrested the growth of cancer cells in the S phase (57.8 vs. 26.8% for UT), along with a notable decrease in the number of cells in both G0/G1 (39.9 vs. 63.1% for UT) and G2/M phases (2.2 vs. 10.0% for UT). The aforementioned treatment also induced apoptosis (cells in sub G1, 14.8 vs. 3.8% for UT; Fig. 6A).

The human CRC cell line SW837 was treated with Sora (5 µM), Cur (200 and 400 µM), and the combination of Sora and Cur was administered in a sequential manner. The effects of these treatments on the cell cycle were monitored using flow cytometry, and the results are presented in Fig. 6B. Treatment with Sora (5 µM) in SW837 cells arrested cancer cell growth in both the S phase (34.9 vs. 27.3% for UT) and G2/M (12.9 vs. 6.1% for UT) phases and reduced the population of cells in the G0/G1 phase (52.0 vs. 66.5% for UT). Treatment with Sora induced apoptosis in SW837 cells at minimal levels (cells in sub G1, 1.4 vs. 1.1% for UT). However, treatment with Cur (200 µM) markedly arrested SW837 cells in the S phase (44.6 vs. 27.3% for UT) but only slightly in G2/M phases (6.2 vs. 6.1% for UT), with a further reduction in the number of cells in the G0/G1 phases (49.1 vs. 66.5% for UT). Treatment with Cur (200 µM) induced apoptosis in SW837 cells (cells in sub G1, 14.7 vs. 1.1% for UT). Sequential treatment with Sora (5 µM) and Cur (200 µM) markedly arrested SW837 cell growth in the S phase (54.9 vs. 27.3% for UT), which was associated with a decreased cell percentage in both G0/G1 (42.2 vs. 66.5% for UT) and G2/M phases (2.7 vs. 6.1% for UT). Moreover, apoptosis was induced following the same combination treatment (cells in sub G1, 4.4 vs. 1.1% for UT). Treatment with Cur (400 µM) arrested SW837 cell growth in the S phase (39.4 vs. 27.3% for UT) with a reduction in cell number in both G0/G1 (58.1 vs. 66.5 for UT) and G2/M phases (2.4 vs. 6.1% for UT). Treatment with Cur (400 µM) induced apoptosis in SW837 cells (cells in sub G1, 2.9 vs. 1.1% for UT). Sequential treatment of SW837 cells with Sora (5 µM) and Cur (400 µM) markedly arrested growth in the S phase (53.6 vs. 27.3% for UT), with a reduction in the population of cells in both G0/G1 (42.2 vs. 66.5% for UT) and G2/M phases (4.0 vs. 6.1% for UT), and an increased level of apoptosis (cells in sub G1, 7.3 vs. 1.1% for UT; Fig. 6B).

**Fig. 7:**
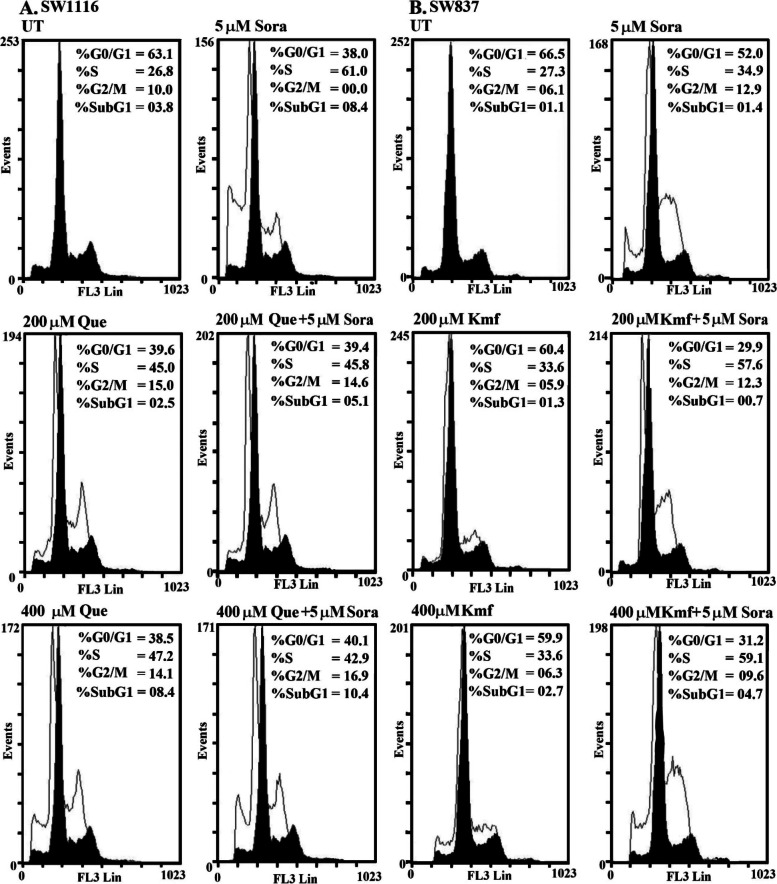
Cell cycle of human CRC cell lines treated with Sora, Que or Kmf and a combination of all treatments. **A** The SW1116 human CRC cell line was UT, or treated with Sora (5 µM), Que (200 µM), Que (400 µM), and simultaneous combinations of Sora and Que (5 + 200 µM or 5 + 400 µM) for 72 h. **B** The SW837 human CRC cell line was UT, or treated with Sora (5 µM), Kmf (200 µM), Kmf (400 µM), and sequential treatments of Sora and Kmf (5 + 200 µM or 5 + 400 µM) for 72 h. Sora, sorafenib; Que, quercetin; Kmf, kaempherol; UT, untreated; CRC, colorectal cancer

Que treatment (200 µM) arrested SW1116 cells in the S phase (45.0 vs. 26.8% for UT) and G2/M phase (15.0 vs. 10.0% for UT), with a decrease in the population of cells in G0/G1 phase (39.6 vs. 63.1% for UT). Simultaneous treatment with Sora (5 µM) and Que (200 µM) also markedly arrested the cells in both the S phase (45.8 vs. 26.8% for UT) and G2/M (14.6 vs. 10.0% for UT), accompanied by a decrease in the percentage of cells in G0/G1 (39.4 vs. 63.1% for UT). The aforementioned treatment also induced apoptosis (cells in sub G1, 5.1 vs. 3.8% for UT; Fig. 7A). Furthermore, Que (400 µM) arrested SW1116 cells in both the S phase (47.2 vs. 26.8% for UT) and G2/M phases (14.1% vs. 10.0% for UT) at high levels, with a corresponding decrease in the population of cells in the G0/G1 phases (38.5 vs. 63.1% for UT). Que (400 µM) also induced SW1116 cell apoptosis, as indicated by an increase in the percentage of cells in the sub G1 phase (8.4 vs. 3.8% for UT). In addition, simultaneous treatment of SW1116 cells with Sora (5 µM) and Que (400 µM) markedly arrested the cells in both the S phase (42.9 vs. 26.8% for UT) and G2/M phases (16.9 vs. 10.0% for UT), with a notable reduction in the population of cells in G0/G1 (40.1 vs. 63.1% for UT). Moreover, apoptosis was markedly induced by the same combination (cells in sub G1, 10.4 vs. 3.8% for UT; Fig. 7A).

Treatment of SW837 cells with Kmf (200 µM) arrested cancer cell growth in the S phase (33.6%vs. 27.3% for UT), with minimal reduction in the population of cells in both G0/G1 (60.4 vs. 66.5% for UT) and G2/M phases (5.9 vs. 6.1% for UT). Kmf (200 µM) also induced apoptosis (cells in sub G1, 1.3 vs. 1.1% for UT). By contrast, sequential treatment with Sora (5 µM) and Kmf (200 µM) markedly arrested the growth of SW837 in both the S phase (57.6 vs. 27.3 for UT) and G2/M phases (12.3 vs. 6.1% for UT). Cells in the sub G1 phase were 0.7 vs 1.1% for UT (Fig. 7B). Treatment with Kmf (400 µM) arrested the growth of SW837 in the S phase (33.6 vs. 27.3% for UT), with an insignificant effect on G2/M phase (6.3 vs. 6.1% for UT) and a decrease in the number of cells in G0/G1 phases (59.9 vs. 66.5% for UT). Kmf (400 µM) also induced apoptosis in SW837 cells (cells in sub G1, 2.7 vs. 1.1% for UT). However, sequential treatment of SW837 cells with Sora (5 µM) and Kmf (400 µM) markedly arrested the cells in the S phase (59.1 vs. 27.3% for UT) and G2/M phase (9.6 vs. 6.1% for UT), with a decrease in the G0/G1 population (31.2 vs. 66.5% for UT) and induction of apoptosis (cells in sub G1, 4.7 vs. 1.1% for UT; Fig. 7B).

### Analysis of DNA fragmentation

**Fig. 8:**
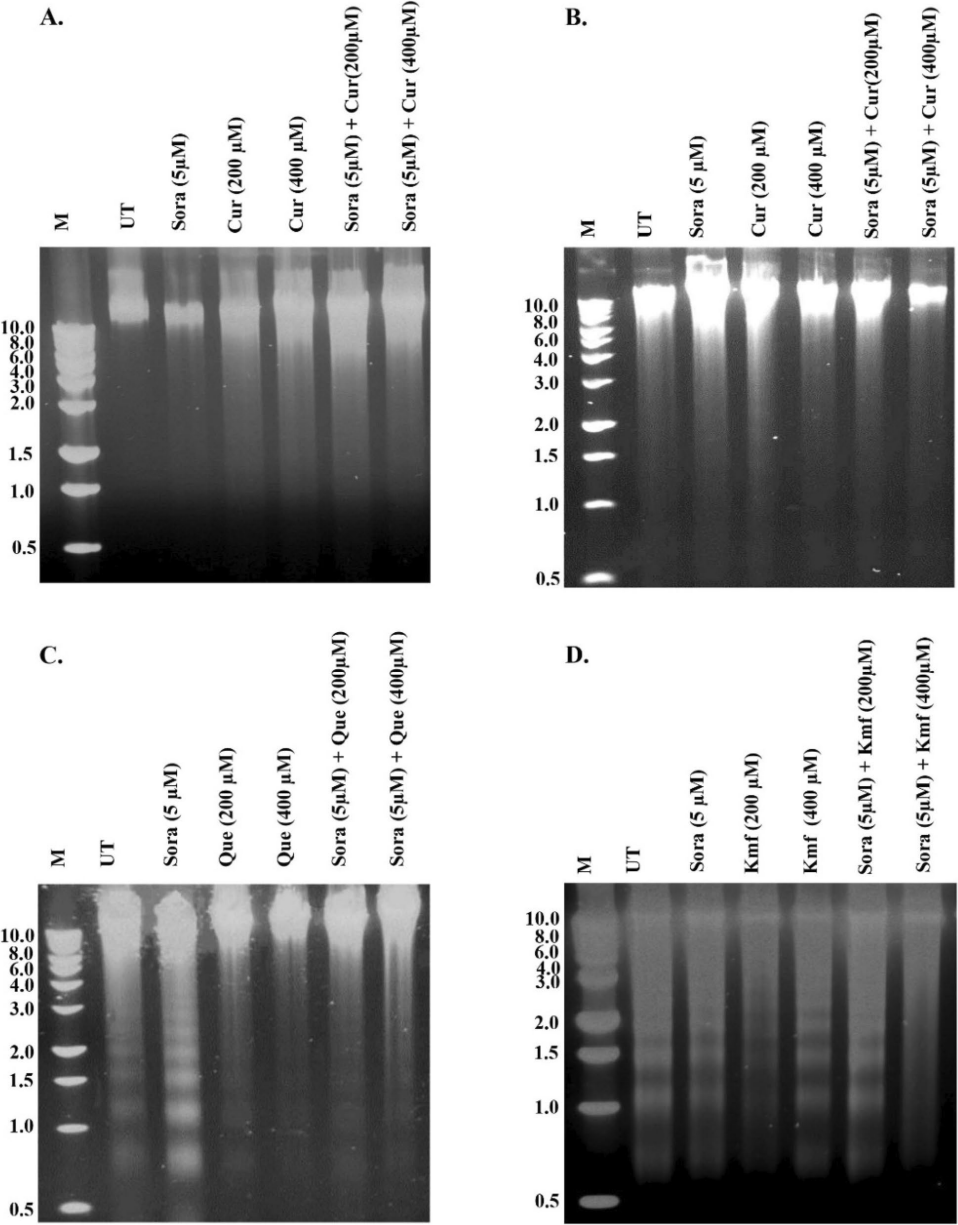
Analysis of DNA fragmentation in human CRC cell lines treated with single and combined treatments of Sora, Cur, Que or Kmf. **A** and **B** SW1116 and SW837 CRC cell lines treated with Sora (5 µM), Cur (200 and 400 µM) and their simultaneous or sequential treatments. **C** SW1116 treated with Sora (5 µM), Que (200 and 400 µM) and their simultaneous treatments. **D** SW837 treated with Sora (5 µM), Kmf (200 and 400 µM), and their sequential treatments for 72 h. M, 1,000 bp DNA marker; Sora, sorafenib; Cur, curcumin; Que, quercetin; Kmf, kaempherol; UT, untreated; CRC, colorectal cancer

DNA fragmentation is one of the hallmarks of apoptosis, in which chromosomal DNA is cleaved into 180–200-bp fragments. Treating the human colon cancer cell line SW1116 with Sor (5 µM), Cur (200 and 400 µM), Que (200 and 400 µM), and simultaneous combination of Sora and either Cur or Que resulted in a marked induction of apoptosis, as indicated by DNA fragmentation, in a dose-, PPC type- and combination-dependent manner (Fig. 8A and C). However, increased levels of DNA fragmentation were detected in the human rectum cancer cell line SW837 incubated with Sora (5 µM), Cur (200 and 400 µM), Kmf (200 and 400 µM) and the sequential combination of Sor and Cur or Kmf in a dose-, PPC type- and combination-dependent pattern (Fig. 8B and D).

#### Annexin V/DAPI double staining

Annexin V/DAPI double staining allows live cells (not stained by either fluorochrome) to be determined from apoptotic cells (stained only by annexin) and necrotic cells (stained by both annexin V and PI).

**Fig. 9:**
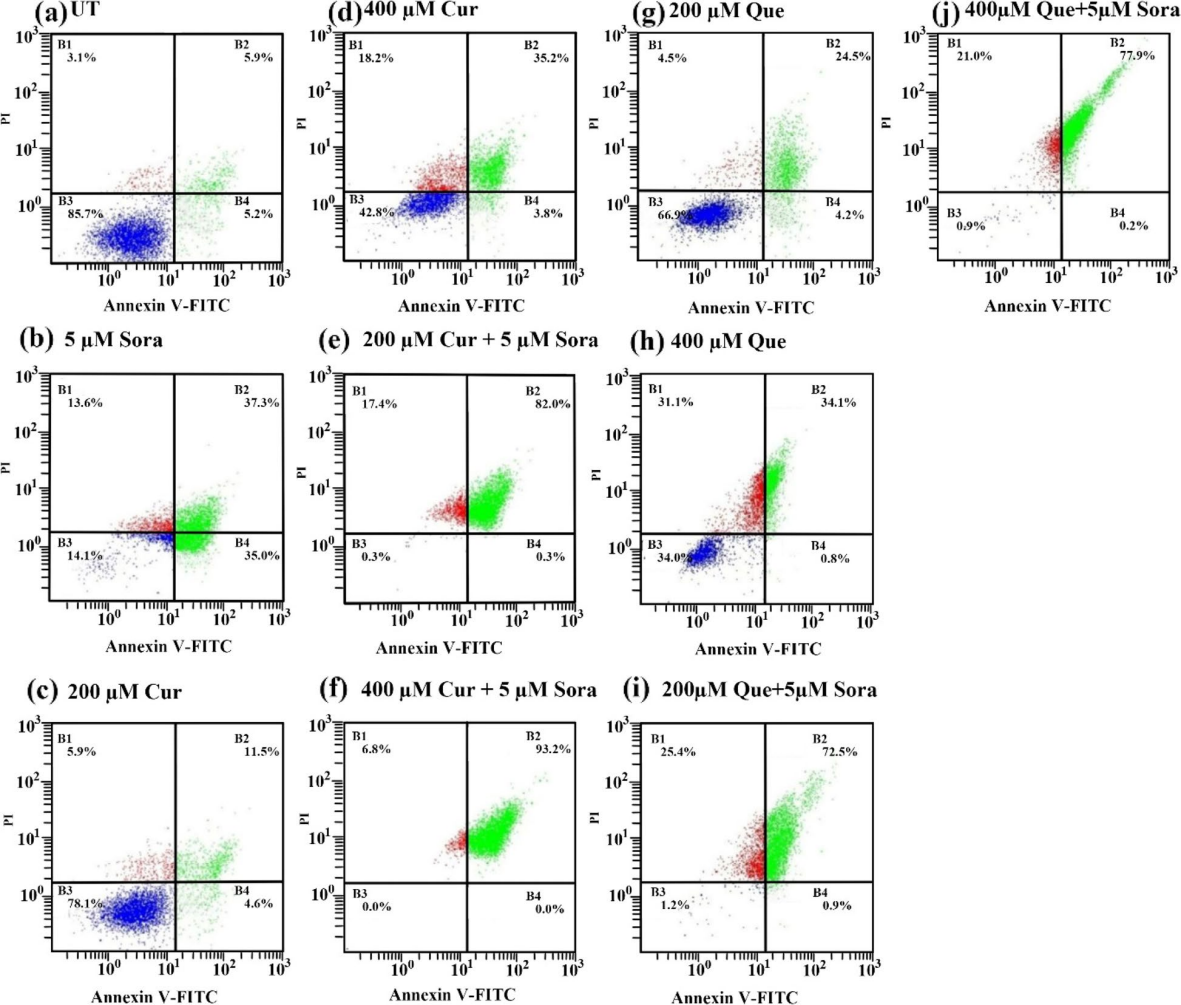
Flow cytometric analysis of apoptosis in human colon cancer cell line SW1116 with Sora, Cur, or Que, and a combination of both. SW1116 was untreated (**a**), treated with Sora (5 µM) (**b**), Cur (200 µM)(**c**), Cur (400 µM) (**d**), and simultaneous combinations of Sora and Cur (5 + 200 µM) (**e**) or (5 + 400 µM) (**f**) or treated with Que (200 µM) (**g**), Que (400 µM) (**h**), and simultaneous combinations of Sora (5 µM) and Que (200 or 400 µM) (**i**, **j**) for 72 h. B1, percentage of necrotic cells; B2, percentage of late apoptotic cells; B3, percentage of viable cells; and B4, percentage of early apoptotic cells. Sora, sorafenib; Cur, curcumin; UT, untreated; PI, propidium iodide; FITC, fluorescein isothiocyanate; CRC, colorectal cancer

The UT human colon cancer cell line SW1116 demonstrated a low level of apoptosis, with 5.2% of the cells exhibiting early apoptosis, 5.9% late apoptosis and 3.1% necrosis (Fig. 9a). However, SW1116 cells treated with Sora (5 µM) demonstrated an increased level of apoptosis, with 35% of the cells exhibiting early apoptosis, 37.3% late apoptosis and 13.6% necrosis (Fig. 9b). Moreover, Cur (200 µM) also induced apoptosis, with 4.6% of cells displaying early apoptosis, 11.5% late apoptosis, and 5.9% necrosis (Fig. 9c); while Cur (400 µM) demonstrated increased levels of apoptosis with 3.8% of cells presenting early apoptosis, 35.2% late apoptosis and 18.2% necrosis (Fig. 9d). The simultaneous combination of Sora (5 µM) and Cur (200 µM) exerted the marked induction of apoptosis, with 0.3% of cells exhibiting early apoptosis, 82.0% late apoptosis, and 17.4% necrosis compared with Sora or Cur (200 or 400 μM) alone (Fig. 9e). Similarly, simultaneous treatment with Sora (5 µM) and Cur (400 µM) greatly induced apoptosis, with 0.0% of cells exhibiting early apoptosis, 93.2% late apoptosis, and 6.8% necrosis compared with a single treatment with Sora or Cur (200 or 400 μM; Fig. 9 f).

Treatment of SW1116 with Que (200 µM) markedly induced apoptosis, with 4.2% of cells exhibiting early apoptosis, 24.5% late apoptosis, and 4.5% necrosis (Fig. 9g). Que (400 µM) also exerted increased levels of apoptosis, with 0.8% of cells exhibiting early apoptosis, 34.1% late apoptosis, and 31.1% necrosis (Fig. 9h). Furthermore, the simultaneous combination of Sora (5 µM) and Que (200 µM) exhibited a marked induction of apoptosis, with 0.9% of cells exhibiting early apoptosis, 72.5% late apoptosis and 25.4% necrosis compared with Sora or Que mono-agent treatments (200 or 400 μM; Fig. 9i). Simultaneous treatment with Sora (5 µM) and Que (400 µM) also displayed a marked induction of apoptosis, with 0.2% of cells exhibiting early apoptosis, 77.9% late apoptosis and 21.0% necrosis compared with single treatment with Sora or Que (200 μM or 400 μM; Fig. 9j).

**Fig. 10:**
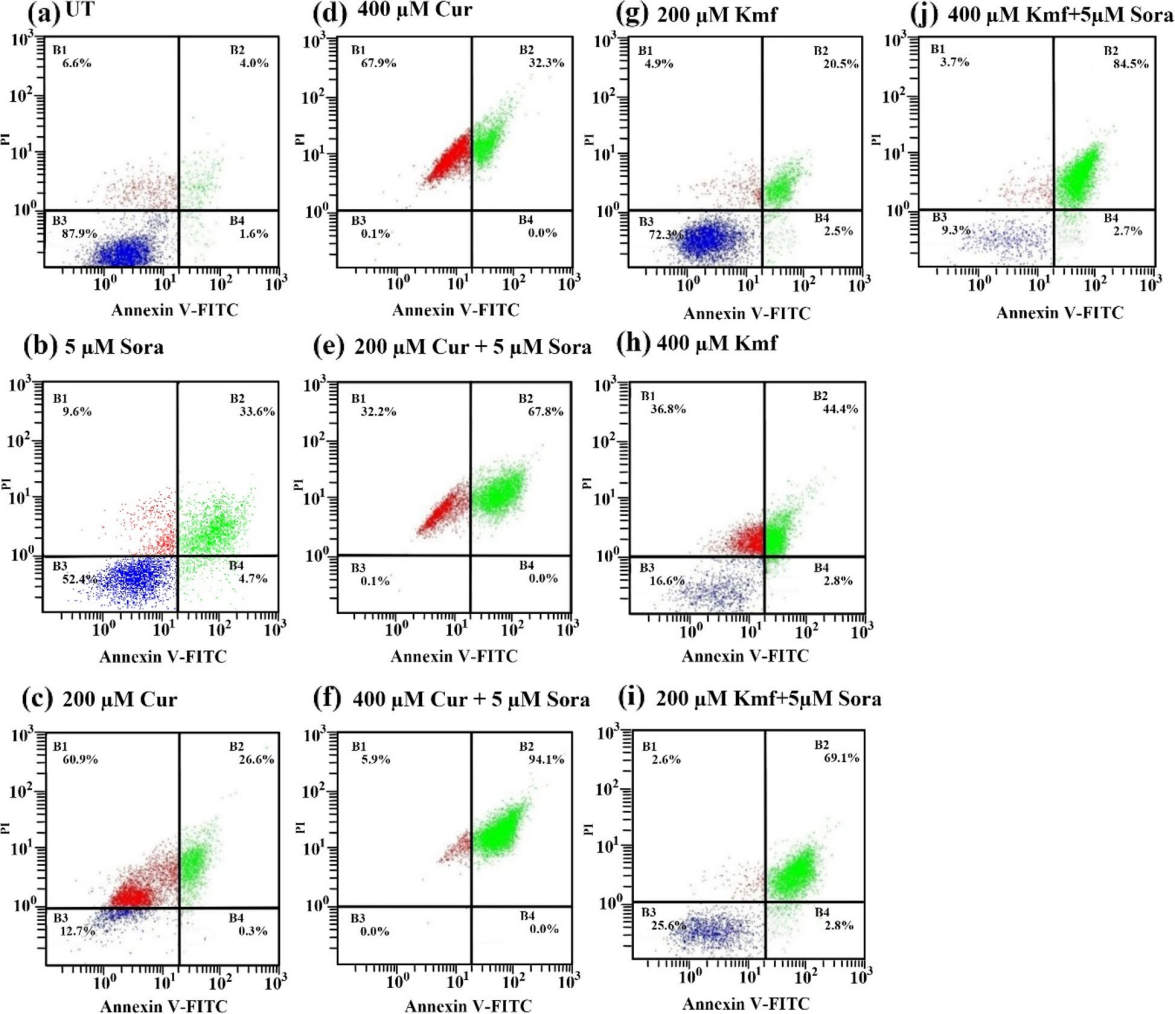
Flow cytometric analysis of apoptosis in human rectum cancer cell line SW837 treated with Sora, Cur, or Kmf and a combination of both. SW837 was untreated (**a**), treated with Sora (5 µM) (**b**), Cur (200 µM) (**c**), Cur (400 µM) (**d**), and sequential treatments of Sora (5 µM) and Cur (200 or 400 µM) (**e**, **f**) or treated Kmf (200 µM) (**g**), Kmf (400 µM) (**h**), and sequential treatments of Sora (5 µM) and Kmf (200 or 400 µM) (**i**, **j**) for 72 h. B1, percentage of necrotic cells; B2, percentage of late apoptotic cells; B3, percentage of viable cells; and B4, percentage of early apoptotic cells. Sora, sorafenib; Que, quercetin; Kmf, kaempherol; UT, untreated; PI, propidium iodide; FITC, fluorescein isothiocyanate; CRC, colorectal cancer

The UT human rectum cancer cell line SW837 displayed a low level of apoptosis, with 1.6% cells exhibiting early apoptosis, 4.0% late apoptosis, and 6.6% necrosis (Fig. 10a). However, Sora (5 µM) induced high levels of apoptosis, with 4.7% of cells exhibiting early apoptosis, 33.6% late apoptosis, and 9.6% necrosis (Fig. 10b). In addition, Cur (200 µM) exhibited high levels of apoptosis, with 0.3% of cells exhibiting early apoptosis, 26.6% late apoptosis, and 60.9% necrosis (Fig. 10c). Cur (400 µM) also exerted a higher level of apoptosis, with 0.0% of cells displaying early apoptosis, 32.3% late apoptosis, and 67.9% necrosis (Fig. 10d). Sequential treatment with Sora (5 µM) and Cur (200 µM) showed marked induction of apoptosis, with 0.0% cells displaying early apoptosis, 67.8% late apoptosis, and 32.2% necrosis (Fig. 10e). Finally, sequential treatment with Sora (5 µM) and Cur (400 µM) markedly induced apoptosis, with 0.0% of cells displaying early apoptosis, 94.1% late apoptosis, and 5.9% necrosis (Fig. 10f).

Treating SW837 with Kmf (200 µM) induced apoptosis, with 2.5% of cells exhibiting early apoptosis, 20.5% exhibiting late apoptosis, and 4.9% exhibiting necrosis (Fig. 10g). However, Kmf (400 µM) exhibited high levels of apoptosis, with 2.8% of cells exhibiting early apoptosis, 44.4% late apoptosis, and 36.8% necrosis (Fig. 10h). By contrast, sequential treatment with Sora (5 µM) and Kmf (200 µM) exerted a marked induction of apoptosis, with 2.8% of cells displaying early apoptosis, 69.1% late apoptosis and 2.6% necrosis compared with Sora or Kmf mono-agent treatments (200 or 400 µM; Fig. 10i). Finally, sequential treatment with Sora (5 µM) and Kmf (400 µM) displayed the highest level of apoptosis, with 2.7% of cells exhibiting early apoptosis, 84.5% late apoptosis, and 3.7% necrosis, compared with the sequential treatment with Sora (5 µM) and Kmf (200 µM), or mono-agent treatment with Sora or Kmf (200 or 400 µM; Fig. 10j).

### MMP analysis

Alterations in the MMP (Δψm) of the SW1116 and SW837 cells were measured following both single and combined treatments with Sora and Cur, and Que or Kmf. In UT cells, the intensity of red fluorescence increased with an accumulation of MitoNIR dye in the mitochondria; however, in apoptotic cells, the intensity of NIR diminished due to the collapse of the MMP. Changes in the MMP of the human colon cancer cell line SW1116 were determined following a single treatment with Sora, Cur, and que, and simultaneous treatment with Sora and Cur, and Sora and que. In addition, MMP was determined in the human rectal cancer cell line SW837 following single treatment with Sora, Cur, and Kmf, and sequential treatment with Sora and Cur, and Sora and Kmf. 

**Fig. 11:**
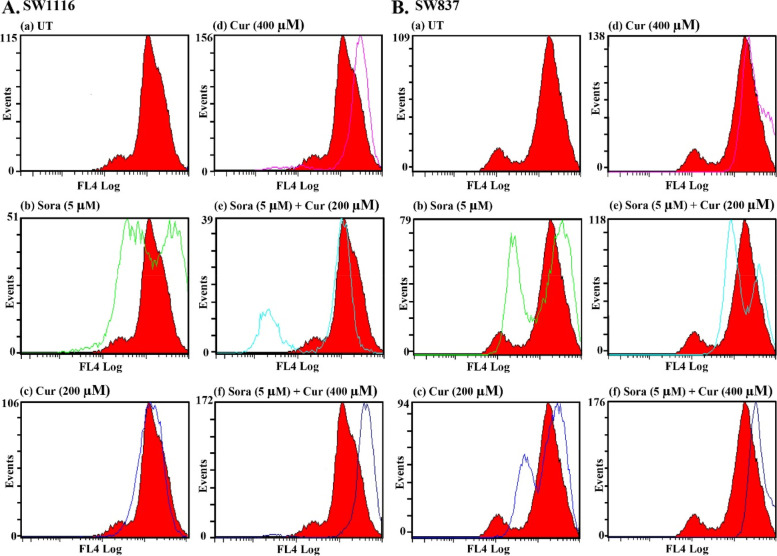
Flow cytometric analysis of MMP in human CRC cell lines treated with Sora, Cur, and a combination of both. **A** The SW1116 human CRC cell line was UT, or treated with Sora (5 µM), Cur (200 µM), Cur (400 µM), and simultaneous combinations of Sora (5 µM) and Cur (200 or 400 µM) for 72 h. **B** The SW837 human CRC cell line was UT, or treated with Sora (5 µM), Cur (200 µM), Cur (400 µM), and sequential treatments of Sora (5 µM) and Cur (200 or 400 µM) for 72 h. The red and white curves are the control and experimental groups, respectively. MMP, mitochondrial membrane potential; Sora, sorafenib; Cur, curcumin; UT, untreated; CRC, colorectal cancer

**Fig. 12:**
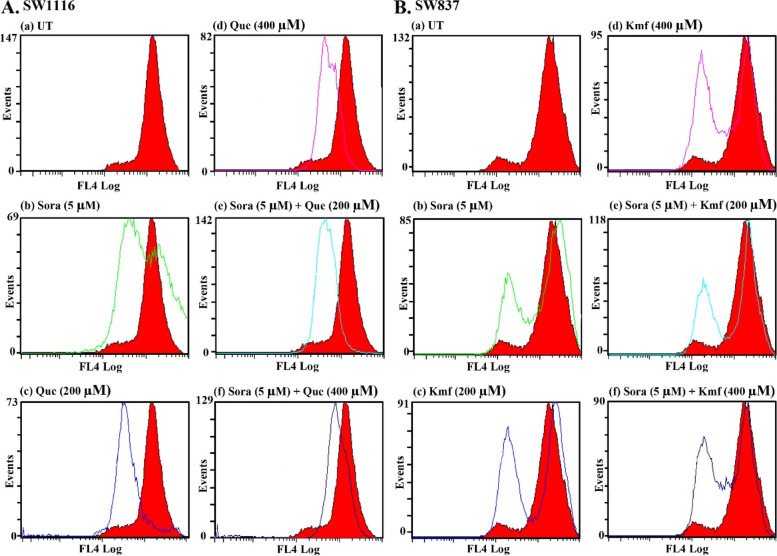
Flow cytometric analysis of MMP in human CRC cell lines treated with Sora, Que or Kmf and a combination of both. **A** The SW1116 human CRC cell line was UT, or treated with Sora (5 µM), Que (200 and 400 µM) and simultaneous combinations of Sora (5 µM) and Que (200 or 400 µM) for 72 h. **B** The SW837 human CRC cell line was UT, or treated with Sora (5 µM), Kmf (200 µM), Kmf (400 µM), and sequential treatments of Sora (5 µM) and Kmf (200 or 400 µM) for 72 h. MMP, mitochondrial membrane potential; Sora, sorafenib; Que, quercetin; Kmf, kaempferol; UT, untreated; CRC, colorectal cancer

Results of the present study indicated that the red fluorescence of the MitoNIR dye had shifted more to the left in combination treatments, compared with treatment with sora or the aforementioned PPCs alone (Figs. 11 and 12). These results suggested a decrease in the intensity of fluorescence, and thus more extensive mitochondrial membrane damage due to more active apoptosis following combined treatment. Moreover, Cur (400 µM) caused a right shift of the red fluorescence, suggesting that there was no change in MMP. Perturbation of MMP in CRC cells was dependent on cancer cell type, PPC-type, and the schedule of the combined treatment.

### Effects of Sora, Cur, and their combination on the expression of cell cycle- and apoptosis-associated proteins in SW1116 cells

**Fig. 13:**
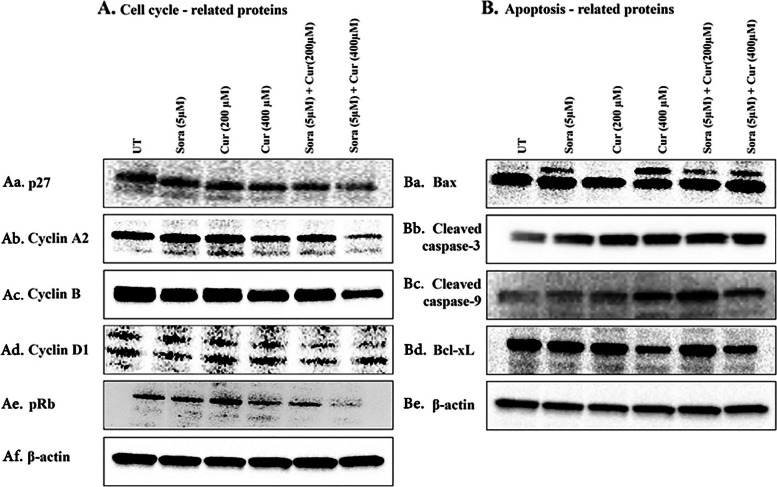
Western blot analysis of the levels of cell cycle and apoptosis-associated proteins in the human colon cancer cell line SW1116 treated with Sora, Cur, and their simultaneous combinations. Cells were treated with Sora (5 µM), Cur (200 µM), Cur (400 µM) or a simultaneous combination of Sora (5 µM) and Cur (200 or 400 µM) for 72 h. The levels of proteins associated with cell cycle and apoptosis control were analysed using western blot analysis. **A** Cell cycle-associated proteins and (**B**) apoptosis-associated proteins. -actin was used as an internal control. Sora, sorafenib; Cur, curcumin; UT, untreated; pRb, phosphor retinoblastoma protein; Bcl-xL, Bcl extra-large protein. Full-length blots/gels are presented in Supplementary Fig. 1 Aa-Af, Ba-Be. Each protein was subjected to two separate western blot analyses. Because of the results' similarity, the WB of every protein for each experiment has been merged into a composite figure and a representative figure was used for publication (Fig. 13 A, B)

The potential mechanism underlying the anticancer effects of Sora, Cur, and their combination on human colon cancer cells were explored by analysing the expression of cell cycle- and apoptosis-associated proteins using western blotting. In SW1116 cells, no significant difference was observed in the expression levels of p27 following combined treatment, compared with the vehicle-treated control (Fig. 13Aa). However, the expression levels of cyclins A2, D1, and pRb were reduced (Figs. 13 Ab, c and e) following simultaneous treatment with Sora and Cur in a dose-dependent manner. However, the expression level of cyclin D did not change following the same combined treatment (Fig. 13Ad). Expression levels of the proapoptotic proteins Bax (Fig. 13Ba), cleaved caspase-3 (Fig. 13Bb), and cleaved caspase-9 (Fig. 13Bc) increased significantly, while the antiapoptotic protein Bcl-xL expression levels (Fig. 13Bd) were decreased following the simultaneous treatment of SW1116 cells with Sora and Cur.β -actin was used as an internal control to ensure the equal loading of protein samples to the gel (Fig. 13Af, Be).

## Discussions

Although Sora has been approved by the Food and Drug Administration for the clinical treatment of HCC and renal cell carcinomas [18], up to 80% of patients develop toxic side effects, such as hand-foot syndrome, diarrhoea, fatigue, rash, and weight loss [19]. The undesirable side effects of sora may demand a dose reduction in > 60% of patients, and even inhibition of treatment in 6–25% of patients [19].

Considerable effort has been devoted to improving the therapeutic efficacy, reducing the debilitating side effects, and overcoming drug resistance associated with sora. Sora elicits a broad spectrum of kinase inhibitory activity, supporting the notion that it may be effective in treating multiple types of cancers, possibly in combination with other cancer therapies. Therefore, a combination therapy that allows a dose reduction of sora, without a concomitant reduction in its efficacy, may prove to be effective in overcoming side effects and evading drug resistance [20].

The present study aimed to investigate the ability of PPCs to potentiate the anticancer effects of sora on human CRC. The optimal combinations were explored, along with the most appropriate administration schedules and the potential underlying molecular mechanisms of action. The combination strategies used in this study are clinically used to test the anticancer drug efficacy [21–23]. We sought to use those clinically applied strategies to test the combination of a clinically used anticancer drug, Sora and PPCs in searching for a novel, more potent combination.

The experimental PPCs used in the present study potentiated the cytotoxicity of Sora on CRC cell lines. Notably, Cur and Kmf exhibited comparable effects, which were more pronounced than the effects of Que, and these effects were more pronounced than those of Rsv, in a dose-, CRC cell line type-, PPCs type- and administration schedule-dependent manner.

The results of the present study are comparable with those obtained from a previous study, demonstrating the use of sora and various anticancer agents for numerous solid tumors [24]. Previous studies have also demonstrated the antitumor effects of the combination of IFN-α and Sora against HCC both in vitro and in vivo [25, 26]. In mice, HCT116 xenograft tumor growth delay experiments indicated that radiation treatment followed by sora was associated with a significant decline in tumor cell growth [27].

Additionally, in C26‑luc metastatic colorectal liver tumor‑bearing mice, the optimum control of tumor growth and survival ratio was attained using combined treatment with 188Re‑liposomes and sora, compared with mono-agent treatment with 188Re-liposomes, and sora or UT normal saline cell groups [28]. Moreover, irinotecan and Sora delayed tumor cell growth in the DLD-1 colon tumor model by 71 and 100%, respectively, while their combination resulted in a 229% synergistic delay. In human colon carcinoma cells, additive or moderate synergistic effects were reported for the combination of Sora and cytotoxic drugs, such as paclitaxel, 5-fluorouracil, and SN-38 (the most active metabolite of CPT-11) [29]. However, Sora was reported to decrease the activity of oxaliplatin and cisplatin on CRC cell lines [30]. Furthermore, in a human colon cancer xenograft mouse model with HT29 tumor cells, combination treatment with Sora and 5-FU was equally.effective as monotherapy, with respect to tumor proliferation [31]. The combination of rapamycin and Sora synergistically inhibited the proliferation of CRC cells; furthermore, simultaneous treatment with rapamycin and Sora inhibited the growth of xenografts from CRC cells with mutations in KRAS and PIK3CA [31].

Sulforaphane (SF), a broccoli-derived isothiocyanate, eliminates pancreatic cancer stem cells (CSCs) by downregulating NFκB activity without the induction of toxic side effects. The combination of sora and SF synergistically inhibited pancreatic CSCs in vitro and markedly induced cell death compared with a single treatment with sora or SF, as indicated by cell morphology, colony, and spheroid formation. A single treatment with sora or SF delayed CSC tumor growth in vivo; however, the combination of SF and sora was more potent and elicited significantly reduced tumor growth. The combination of Sora and Que elicited a synergistic antiproliferative effect on human HCC cell lines (HepG2, Huh7, and Hep4B2.1) [32]. This combination also caused effective cell-specific cell death in anaplastic astrocytoma cells, with an increased percentage of dead cells compared with that observed following treatment with sora alone [33]. Combined treatment with Sora and Nanocurcumin produced a more potent antitumor effect on HCC cells, compared with Sora or Nanocurcumin alone. This combination also inhibited HCC cell migration and invasion, while it promoted apoptosis both in vitro and in vivo [34]. Furthermore, Sora curcumin nanoparticles (SCNs) elicited superior effects compared with Sora alone, Cur alone, or a mixture of Sora and Cur, on enhancing the in vitro cytotoxicity of the BEL-7402 and HepG2 HCC cell lines [34]. In HCC BEL7402 cell-induced tumor xenografts, SCN treatment elicited a higher inhibitory effect on tumor progression compared with monotherapy, or a physical mixture of Sora and Cur, with significantly higher antiproliferative and antiangiogenic capabilities [35]. The combination of Sora and YC-1 synergistically induced apoptosis and inhibited the proliferation of HCC cell lines BEL-7402 and HepG2 compared with Sora or YC-1 mono-agent treatment. This combination also markedly suppressed tumor growth in HepG2 xenografts in nude mice, compared with either drug alone [36].

Results of a previous study demonstrated that Cur, Que, Kmf, and Rsv markedly potentiated the therapeutic efficacy of Sora in HCC cell lines in an administration-dependent manner [17]. Moreover, Cur enhanced the antitumor efficacy of Sora in H22-bearing mice treated with the combination of Cur and Sora by activating immune function, downregulating epithelial-mesenchymal transition, and reversing metabolic disorders [37]. Kaempherol sensitized HepG2 and N1S1 to the subtoxic concentration of Sora [38].

The inhibitory effects of various combination treatments using sora and selected PPCs were investigated in vitro in the present study. The results indicated that simultaneous treatments with sora and cur or Sora and que were more effective on SW1116 human colon cancer cells, while sequential treatments with sora and cur or sora, and Kmf were more effective on SW837 human rectal cancer cells.

Cell growth and proliferation is controlled by the cell cycle, and the disparity between proliferation and apoptosis, caused by the disruption of the cell cycle may lead to cancer growth. Thus, anticancer agents that target the cell cycle may arrest the uncontrolled proliferation of cancer cells and promote cell death and apoptosis [39].

Several genetically defined checkpoints control the cell cycle by ensuring the coordinated progression of the cell through the various stages of the cell cycle and monitoring DNA integrity [40].

In the present study, analysis of both the cell cycle and apoptosis indicated that combined treatment of human CRC cells with Sora and Cur, sora and que or sora, and Kmf resulted in growth arrest in the S phase and/or M phase, depending on the cancer type and treatment schedule. The treatments also resulted in the accumulation of cells in the sub G1 phase, which is considered a sign of apoptosis [41]. Moreover, most anticancer drugs induce apoptosis by arresting cells in sub-G1 [42]. Results of the present study are comparable with those previously investigated the combination of INF-λ3 and Sora, which arrested the liver cancer cell lines HepG2 and SMMC7721 in the S phase [43], and simultaneous combined treatment with sora and either Cur or Kmf, which arrested the human HCC cell lines Hep3b and HepG2 in the S phase and G2/M phases [36].

Disruption of the cell cycle in the S phase and G2/M suggests that these combinations may affect DNA synthesis and disrupt cell cycle progression beyond the S phase, thus leading to apoptosis. Moreover, arresting damaged cells in G2/M allows adequate time for DNA damage repair or permanent obstruction of the aberrant cells [44]. Many anticancer drugs induce cell death and apoptosis by arresting the cell cycle in G2/M [45]. Cell cycle arrest at G2/M disturbs the tubulin-microtubule equilibrium [46], suggesting a role for G2/M arrest in inhibiting microtubule dynamics.

Induction of apoptosis is considered expedient in the prevention of cancer [47]. However, a major challenge in cancer treatment is that cancer cells are capable of evade apoptosis. As a safe-guarding mechanism against tumorigenesis, and a consequence of genetic and epigenetic alterations, cancer cells can become resistant to cell death and apoptosis, rendering anticancer drugs ineffective [48].

In the present study, DNA fragmentation, Annexin-V/PI double staining, and monitoring of MMP were carried out to examine the induction of apoptosis by Sora, tested PPCs and their combinations in CRC cell lines. Results of the present study demonstrated that the apoptotic effects of the single and combination treatments depend on the PPC type, the sora/PPC combination, and the treatment schedule. These results are comparable with those reported for several types of tumors treated with various combinations of sora [33–35]. In HCC-bearing mice, the combination of resveratrol and Sora significantly inhibited growth and induced apoptosis, compared with Sora treatment alone [49].

Combination treatment of the liver cancer cell lines HepG2 and SMMC7721 with IFN-λ3 and Sora promoted the loss of MMP and induced reactive oxygen species production more effectively than the monotreatment with either component [43]. Simultaneous treatment of the HCC cell lines HepG2 and Hep3b with Sora and Cur or Kmf resulted in a pronounced induction of apoptosis, compared with the monotreatment with Sora, Cur, or Kmf [36].

Several therapeutic agents induce cancer cell growth arrest and apoptosis by disrupting cell cycle regulation and impairing checkpoint control [50]. Previous evidence suggests that cyclin-dependent kinase inhibitors modulate cyclin/cyclin-dependent kinase complexes; the inhibitors are thought to regulate cell cycle checkpoints, leading to the ultimate cell cycle arrest [51]. To investigate the mechanisms underlying the effects of Sora, Cur, and their combination, the expression of proteins associated with cell cycle and apoptosis was determined using western blotting.

In the present study, western blotting revealed no significant difference in the expression levels of the cyclin-dependent kinase inhibitor p27 and cyclin D1 in SW1116 cells treated simultaneously with Sora, and cur. Cyclin D1 is essential for G1 cell cycle progression, and the subsequent inhibition of its expression would arrest the cells at the G1/S phase [52]. However, the expression levels of cyclins A2, cyclin B, and pRb were reduced in SW1116 cells following the same treatment.

Sora was reported to suppress the growth of a renal carcinoma cell line and renal carcinoma cell-induced xenografts and downregulated the expression of cyclins D1 and B1 [52]. Results of a previous study using HCC cell lines (Hep3B, HLF, HLE, PLC/PRF/5, and Huh-7) and the hepatoblastoma cell line Huh-6 treated with Sora, revealed that Sora induced the downregulation of cyclin D1 expression, and induced cell cycle suppression and apoptosis [53]. Moreover, treatment of Huh-7 with a nano-micelle Cur in combination with Sora reduced cyclin D1 gene expression [54].

Furthermore, treatment of the CRC cell line HT-29 with the combination of Sora and radiation enhanced the cytotoxic effects of Sora, while Sora alone induced tumor cell accumulation in G2/M phase, and decreased cyclin B1 expression [55]. These findings are consistent with our earlier report, which revealed that simultaneous treatment with Sora and Cur reduced the protein levels of cyclins A, B2, and D1, as well as pRb in hepatic cancer cell lines [36].

Induction of apoptosis is regarded as a novel approach to cancer therapy [56], and many anticancer drugs induce tumor cell death by triggering apoptosis [57]. Results of the western blot analysis carried out in the present study demonstrated a pronounced increase in the expression of the proapoptotic proteins Bax, cleaved caspase-3 and cleaved caspase-9, and a decrease in the expression of the anti-apoptotic protein BcL-xL, following the simultaneous treatment of SW1116 cells. An increase in the Bax/BcL-xL ratio results in the collapse of MMP, the release of cytochrome c, activation of caspase-3, and apoptosis [58]. Furthermore, the proapoptotic protein Bax controls the permeability of the mitochondrial membrane and the release of cytochrome c [59]. Treating HCC cell lines and HCC xenografts with Sora induced proteolytic activation of caspase-9 and caspase-3, suggesting that Sora can trigger mitochondrial-mediated apoptosis [60]. Sora prompted caspase-dependent BcL-xL degradation, destabilized the mitochondria, and induced rapid apoptosis in myeloma cells [61]. Results of the present study are comparable with those of a previous study, which suggested that combined treatment of the liver cancer cell lines SMMC7721 and HepG2 with sora and berberine upregulated the expression of the cleaved poly (ADP-ribose) polymerase and cleaved caspase-3, while it downregulated the expression of the antiapoptotic protein cell lympho-2 and VEGF [39]. Simultaneous treatment with Sora and Cur also increased the expression of cleaved caspase-3, cleaved caspase-9, and Bcl-xL in the HepG2 and Hep3b hepatocellular cancer cell lines [34].

The synergistic inhibition of human CRC cell growth by combined treatment with PPCs and sora may be explained by the cascade pathways targeted by sora and PPCs. Results of previous studies have demonstrated that the MAPK signalling cascade is a common target for anticancer agents. Furthermore, Sora and PPCs used in the present study each inhibit other growth regulatory signalling pathways and synergize to exert their antimitogenic effects on CRC cell lines.

## Conclusion

The synergistic effect of PPCs indicates that these agents may act as an adjunct to sora treatment in a sequence-dependent manner of administration, thus reducing its dose-dependent side effects. More detailed investigations of the molecular mechanism and in vivo studies using the animal model are imperative to evaluate whether the combination of sora and natural bioactive compounds could provide a more effective strategy in CRC therapy. Further investigations are also required to determine the effects in other types of cancer.

## Supplementary Information

**Additional file 1. **

## Data Availability

All the data generated and analyzed in this study are mentioned in this manuscript.
