# Supplementary material for: Therapeutic efficacy of sorafenib and plant-derived phytochemicals in human colorectal cancer cells
Source: BMC Complement Med Ther. 2023 Jun 26;23:210. doi: 10.1186/s12906-023-04032-6 (PMC10294390; doi:10.1186/s12906-023-04032-6)
Supplement: Supplementary file 1 — Additional file 1. [file 12906_2023_4032_MOESM1_ESM.zip › Fig 1_Suppl_ Duplicate_Amended_ 28-5-23.pptx]

## Slide 1
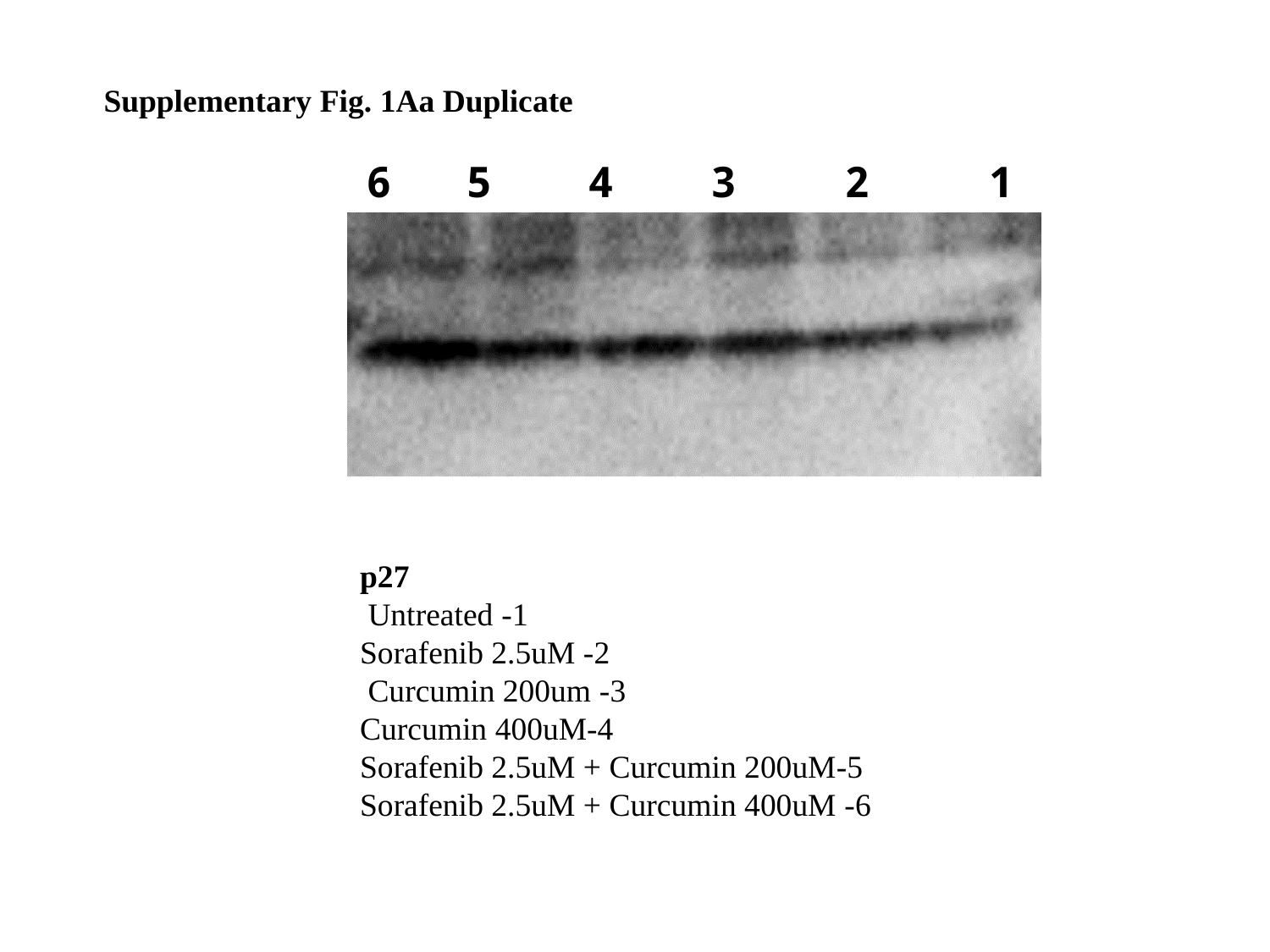

Supplementary Fig. 1Aa Duplicate
1 2 3 4 5 6
p27
1- Untreated
2- Sorafenib 2.5uM
3- Curcumin 200um
4-Curcumin 400uM
5-Sorafenib 2.5uM + Curcumin 200uM
6- Sorafenib 2.5uM + Curcumin 400uM

## Slide 2
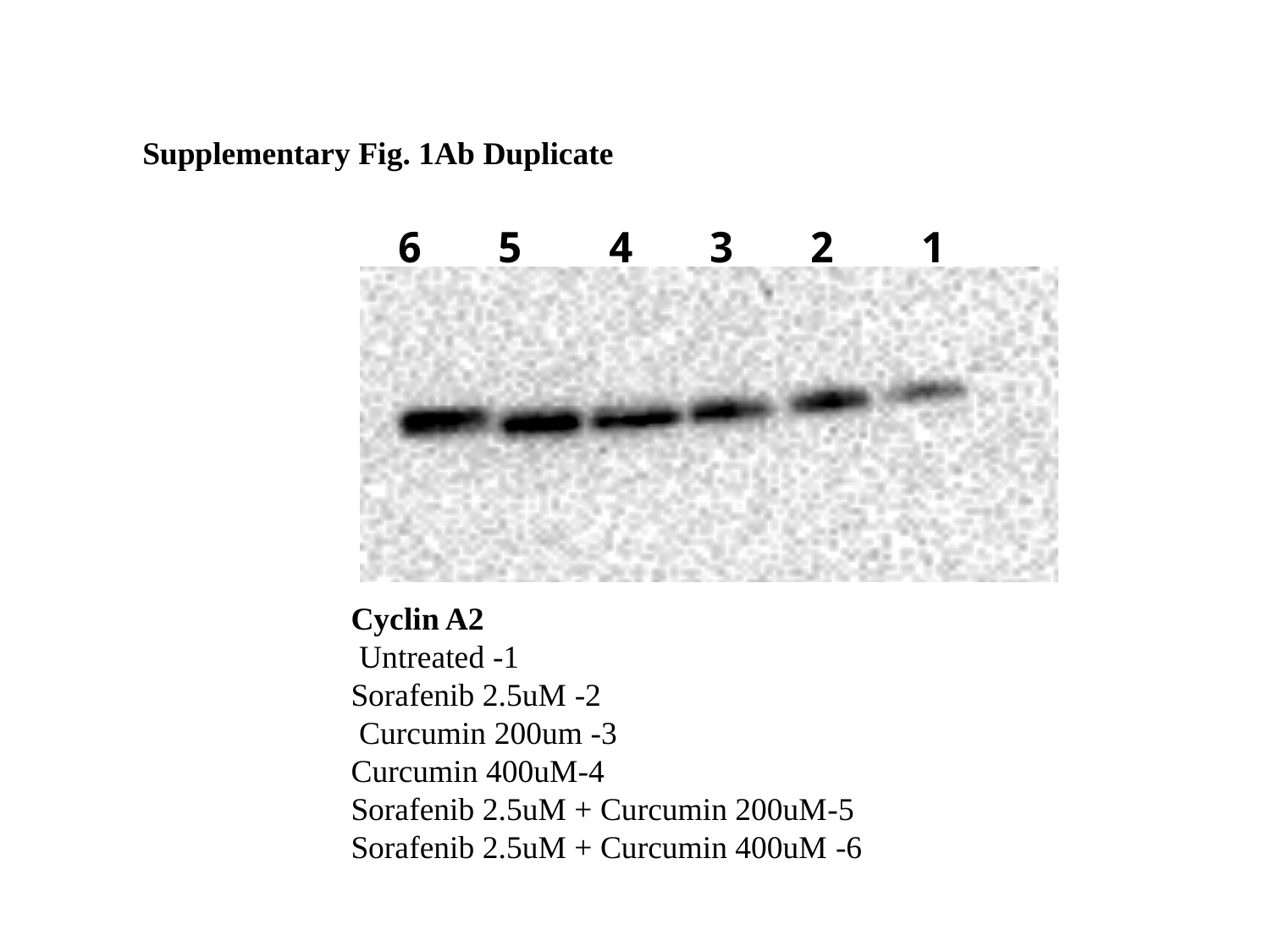

Supplementary Fig. 1Ab Duplicate
1 2 3 4 5 6
Cyclin A2
1- Untreated
2- Sorafenib 2.5uM
3- Curcumin 200um
4-Curcumin 400uM
5-Sorafenib 2.5uM + Curcumin 200uM
6- Sorafenib 2.5uM + Curcumin 400uM

## Slide 3
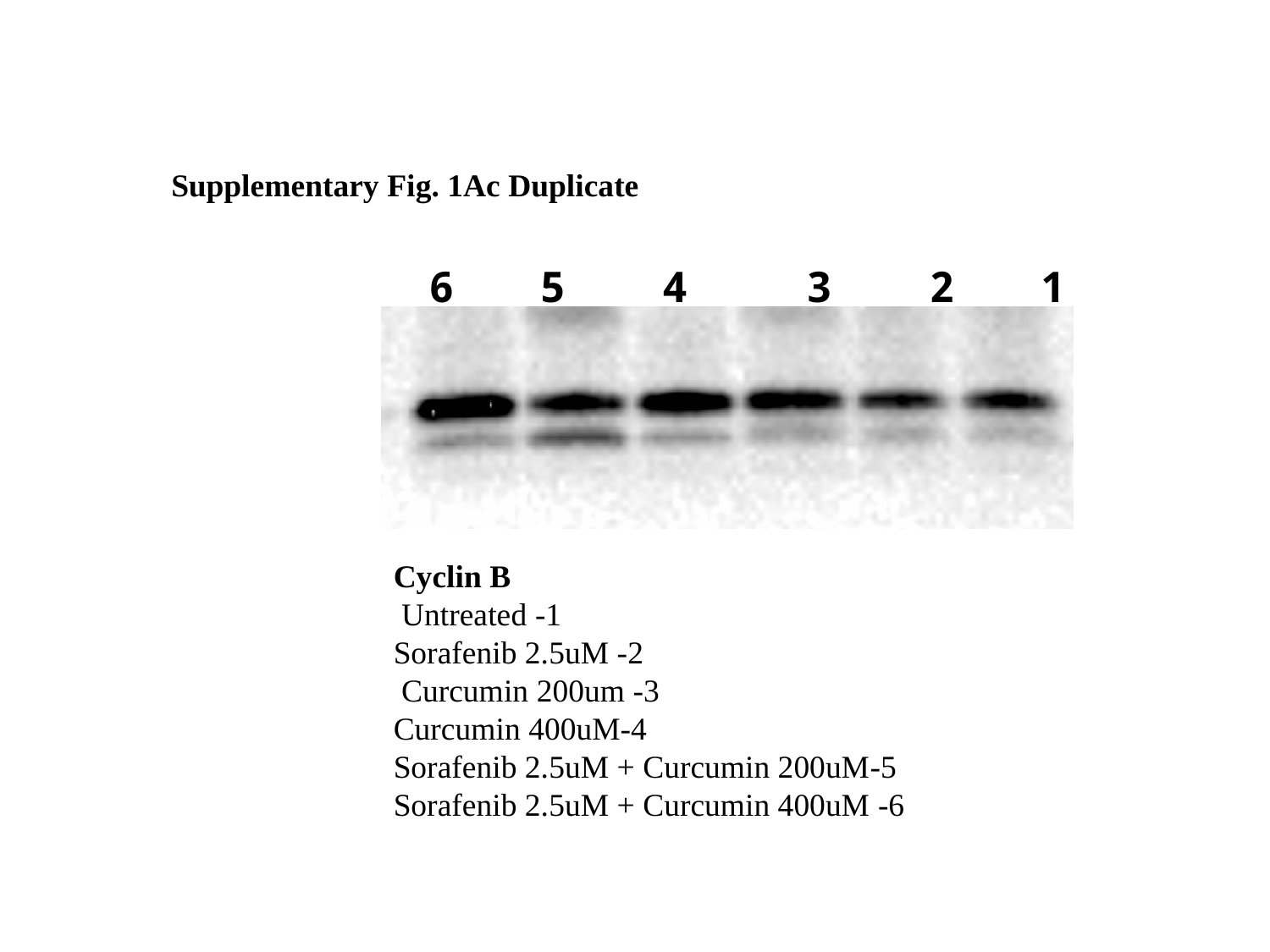

Supplementary Fig. 1Ac Duplicate
1 2 3 4 5 6
Cyclin B
1- Untreated
2- Sorafenib 2.5uM
3- Curcumin 200um
4-Curcumin 400uM
5-Sorafenib 2.5uM + Curcumin 200uM
6- Sorafenib 2.5uM + Curcumin 400uM

## Slide 4
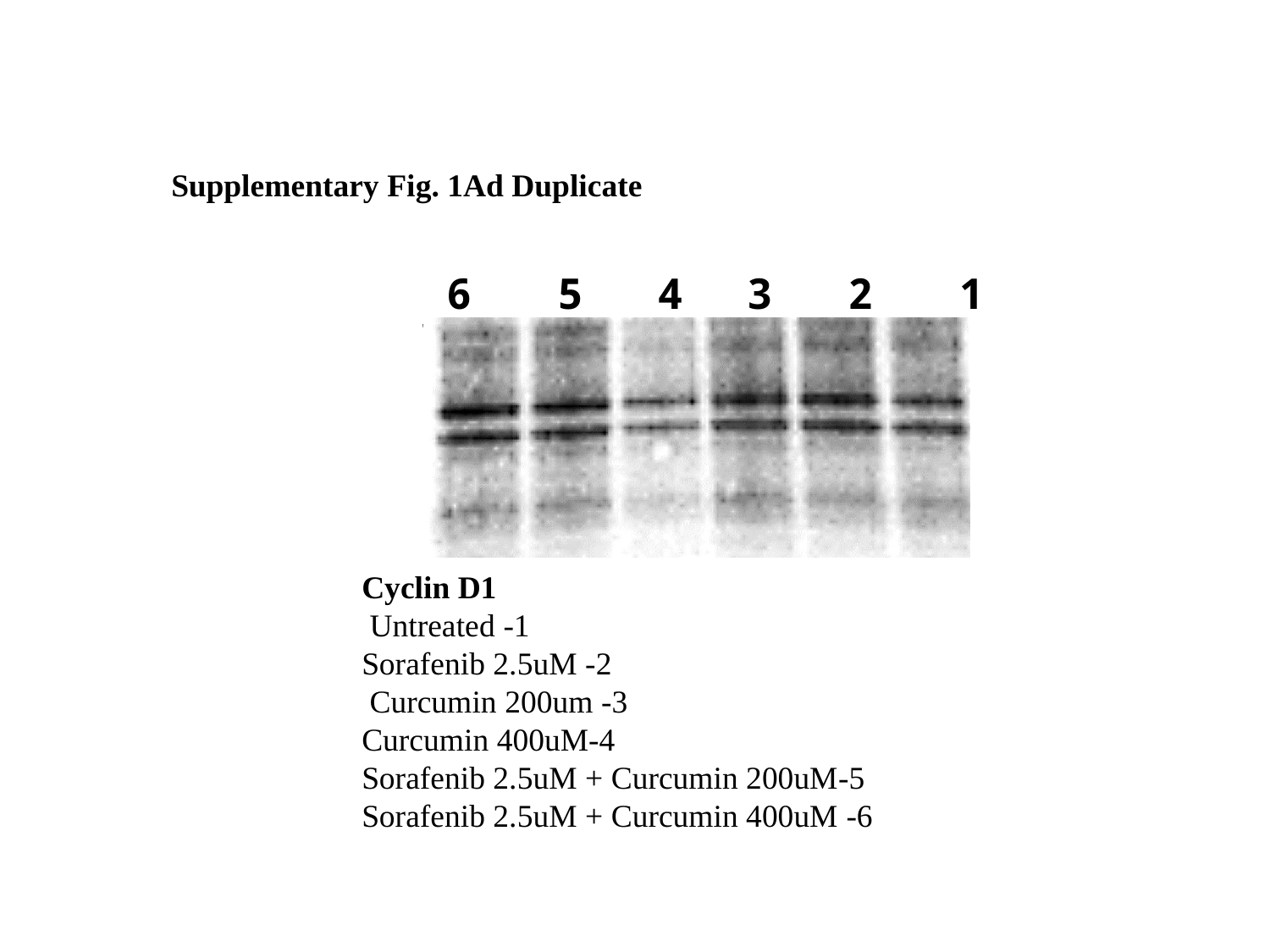

Supplementary Fig. 1Ad Duplicate
1 2 3 4 5 6
Cyclin D1
1- Untreated
2- Sorafenib 2.5uM
3- Curcumin 200um
4-Curcumin 400uM
5-Sorafenib 2.5uM + Curcumin 200uM
6- Sorafenib 2.5uM + Curcumin 400uM

## Slide 5
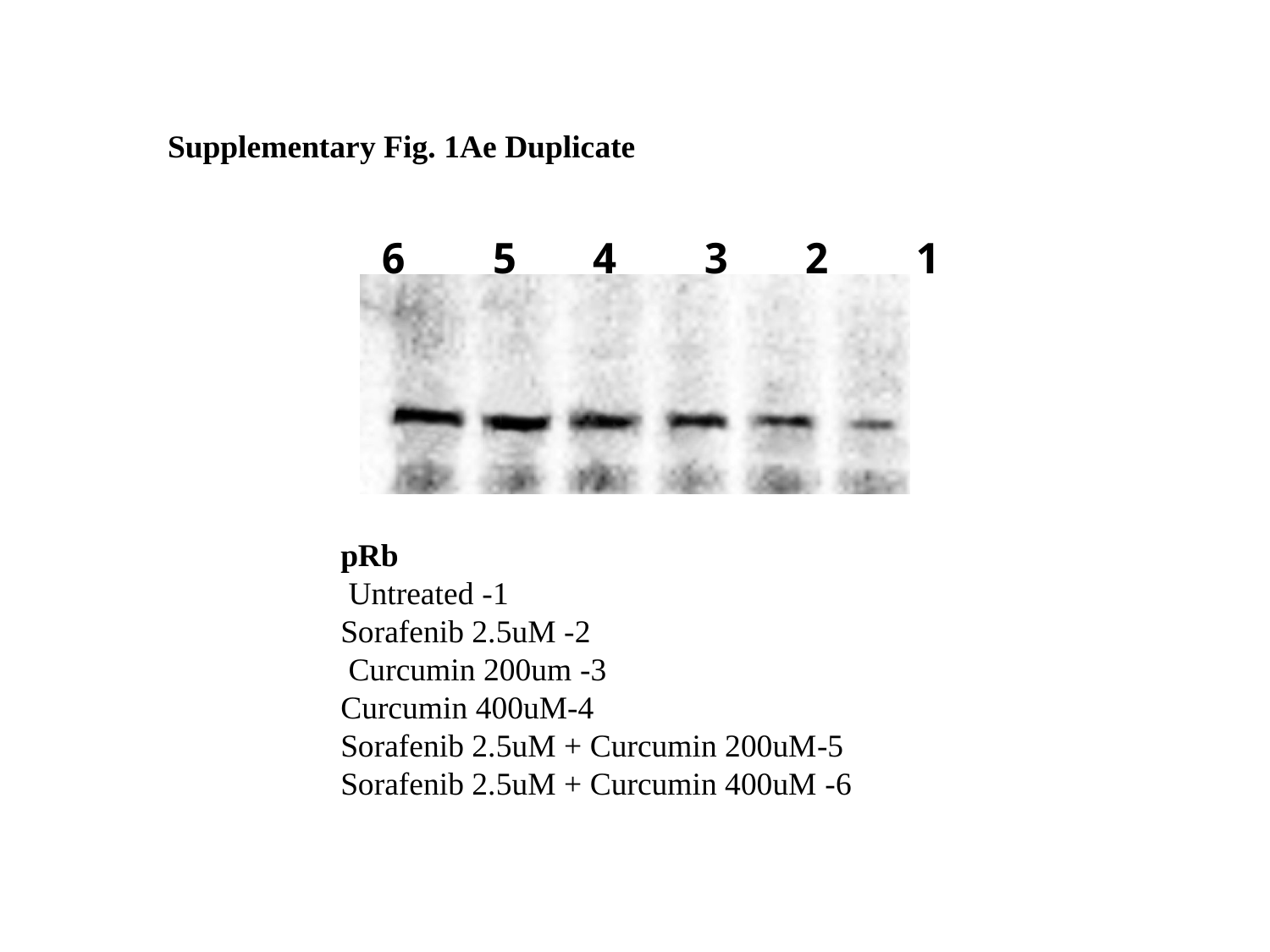

Supplementary Fig. 1Ae Duplicate
1 2 3 4 5 6
pRb
1- Untreated
2- Sorafenib 2.5uM
3- Curcumin 200um
4-Curcumin 400uM
5-Sorafenib 2.5uM + Curcumin 200uM
6- Sorafenib 2.5uM + Curcumin 400uM

## Slide 6
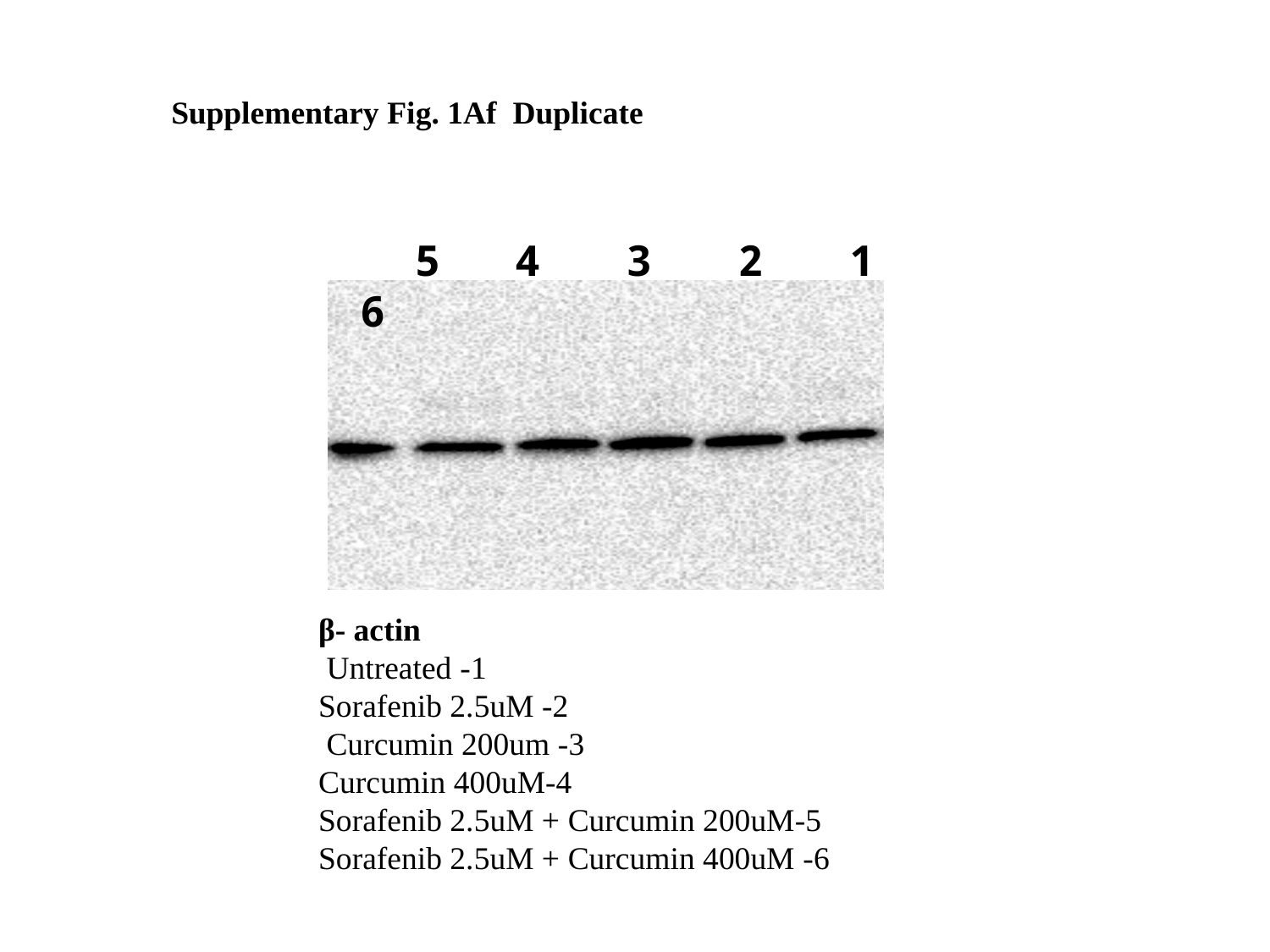

Supplementary Fig. 1Af Duplicate
1 2 3 4 5 6
β- actin
1- Untreated
2- Sorafenib 2.5uM
3- Curcumin 200um
4-Curcumin 400uM
5-Sorafenib 2.5uM + Curcumin 200uM
6- Sorafenib 2.5uM + Curcumin 400uM

## Slide 7
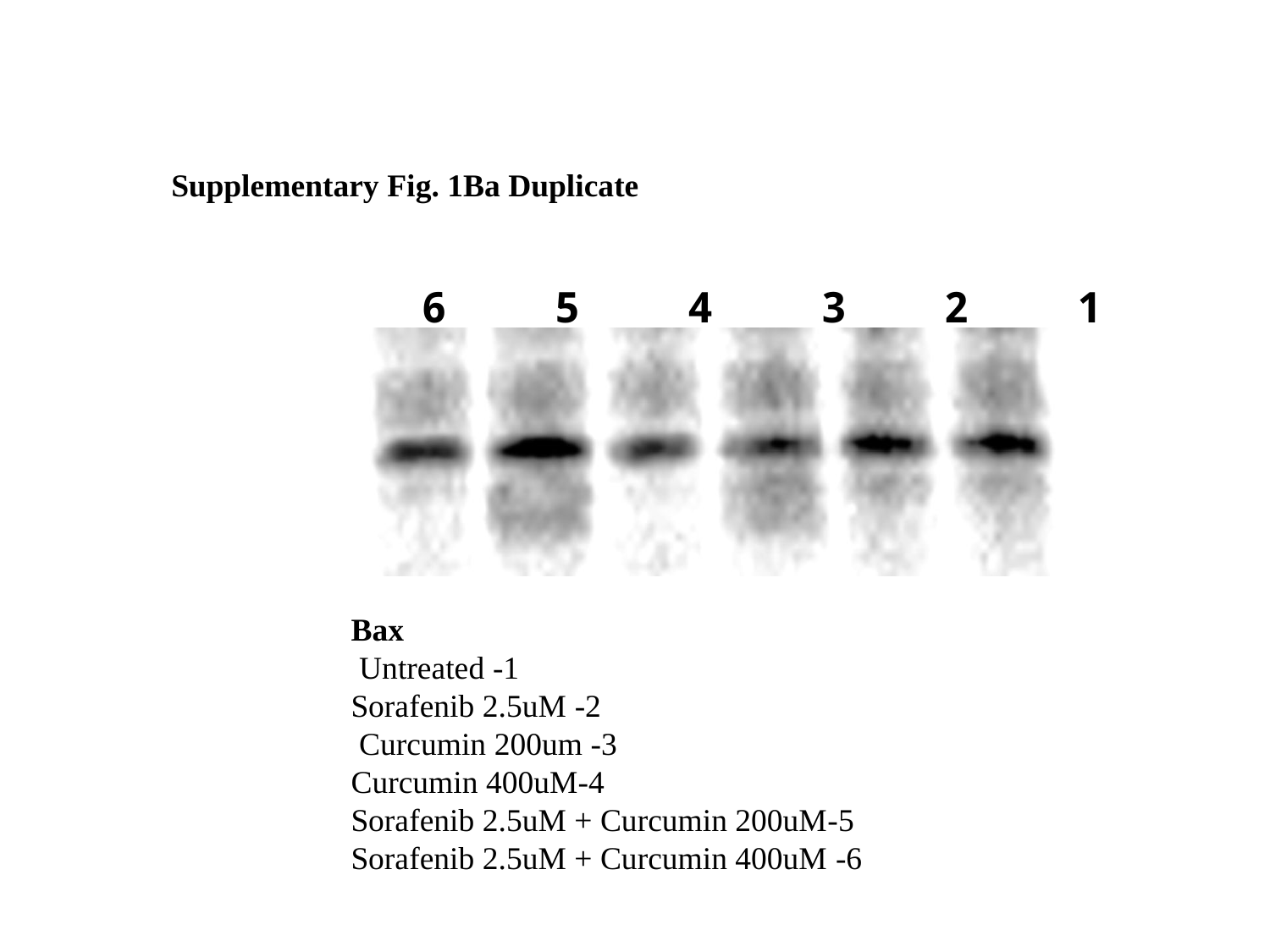

Supplementary Fig. 1Ba Duplicate
1 2 3 4 5 6
Bax
1- Untreated
2- Sorafenib 2.5uM
3- Curcumin 200um
4-Curcumin 400uM
5-Sorafenib 2.5uM + Curcumin 200uM
6- Sorafenib 2.5uM + Curcumin 400uM

## Slide 8
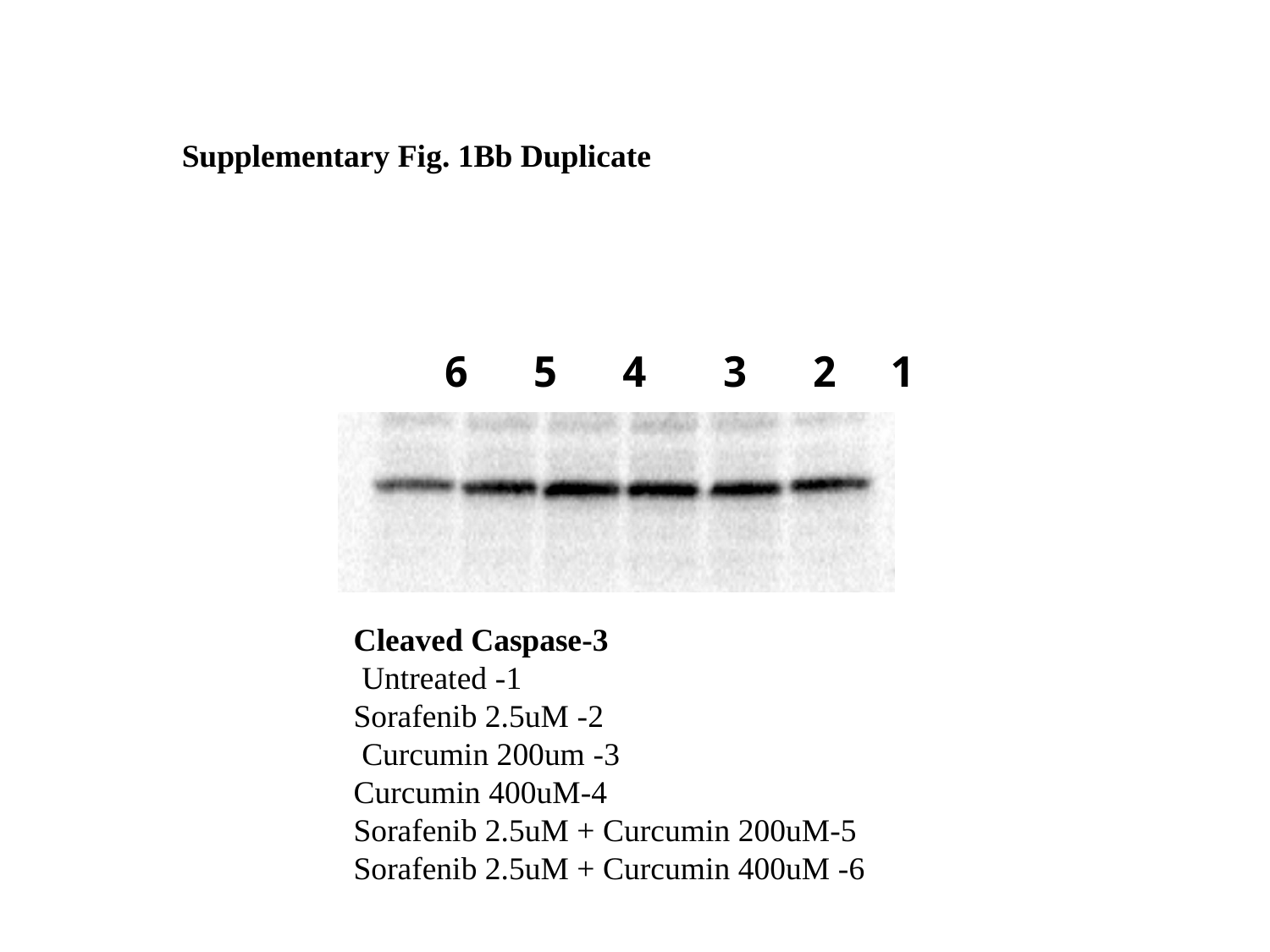

Supplementary Fig. 1Bb Duplicate
1 2 3 4 5 6
Cleaved Caspase-3
1- Untreated
2- Sorafenib 2.5uM
3- Curcumin 200um
4-Curcumin 400uM
5-Sorafenib 2.5uM + Curcumin 200uM
6- Sorafenib 2.5uM + Curcumin 400uM

## Slide 9
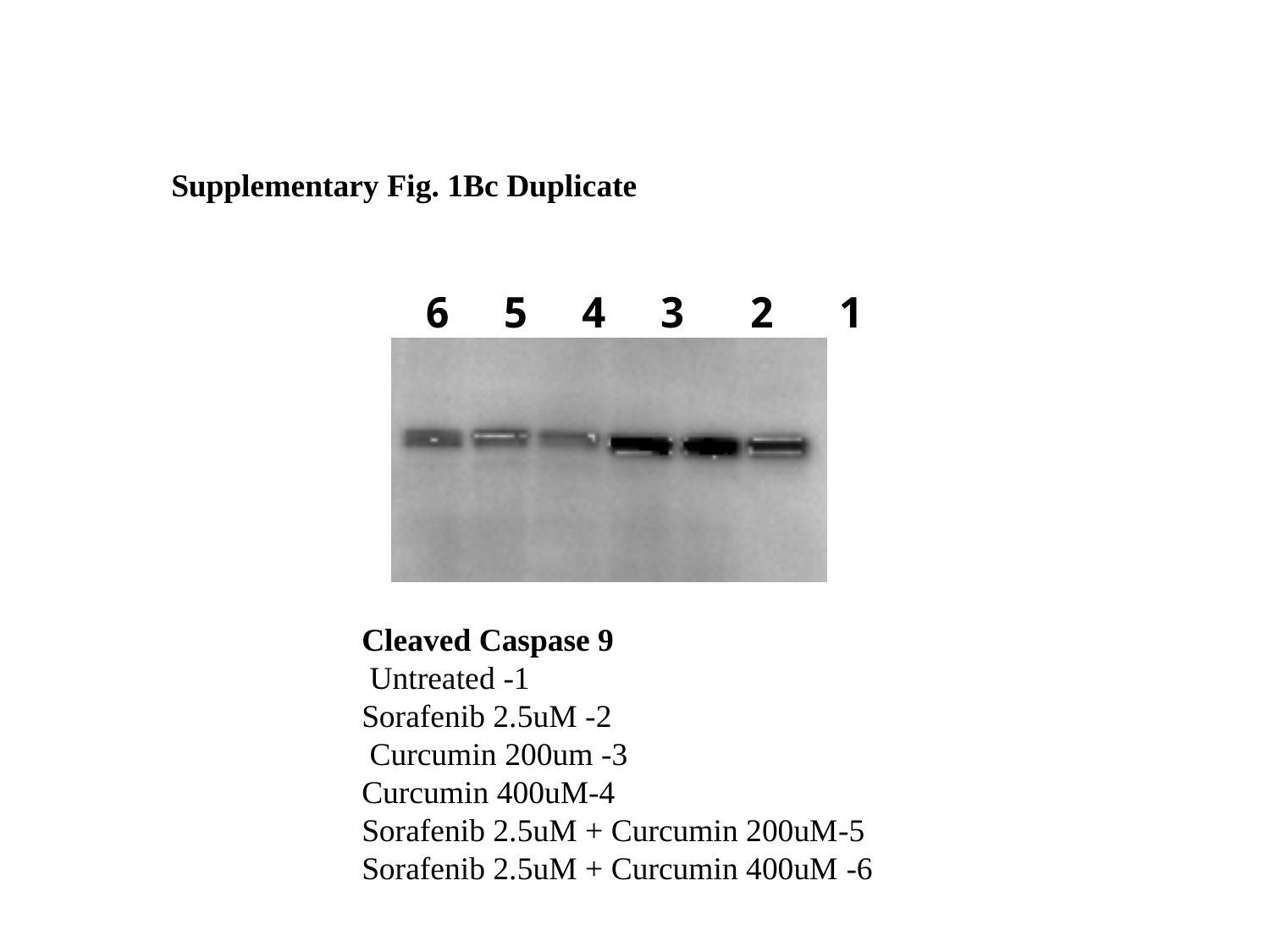

Supplementary Fig. 1Bc Duplicate
1 2 3 4 5 6
Cleaved Caspase 9
1- Untreated
2- Sorafenib 2.5uM
3- Curcumin 200um
4-Curcumin 400uM
5-Sorafenib 2.5uM + Curcumin 200uM
6- Sorafenib 2.5uM + Curcumin 400uM

## Slide 10
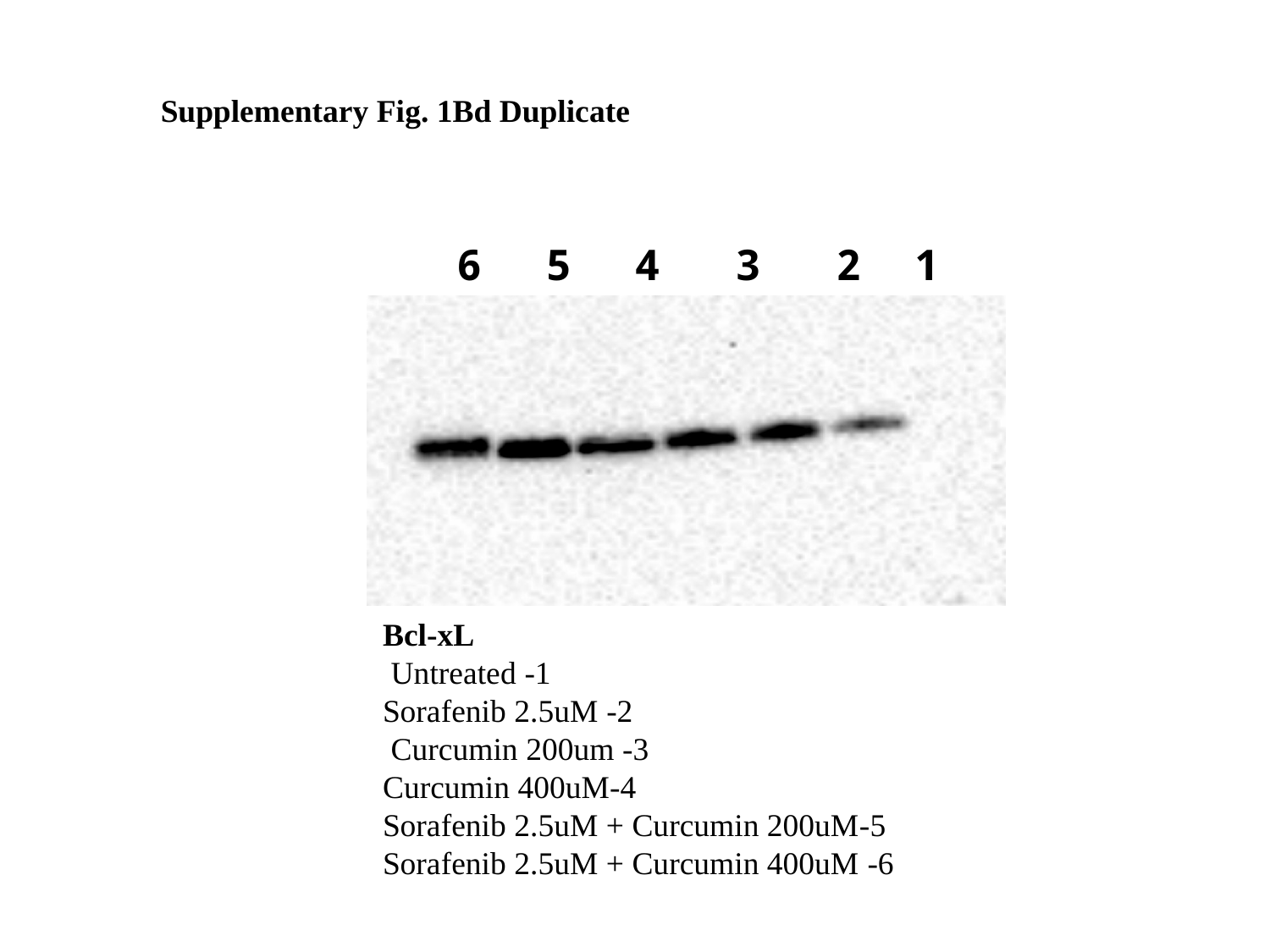

Supplementary Fig. 1Bd Duplicate
1 2 3 4 5 6
Bcl-xL
1- Untreated
2- Sorafenib 2.5uM
3- Curcumin 200um
4-Curcumin 400uM
5-Sorafenib 2.5uM + Curcumin 200uM
6- Sorafenib 2.5uM + Curcumin 400uM

## Slide 11
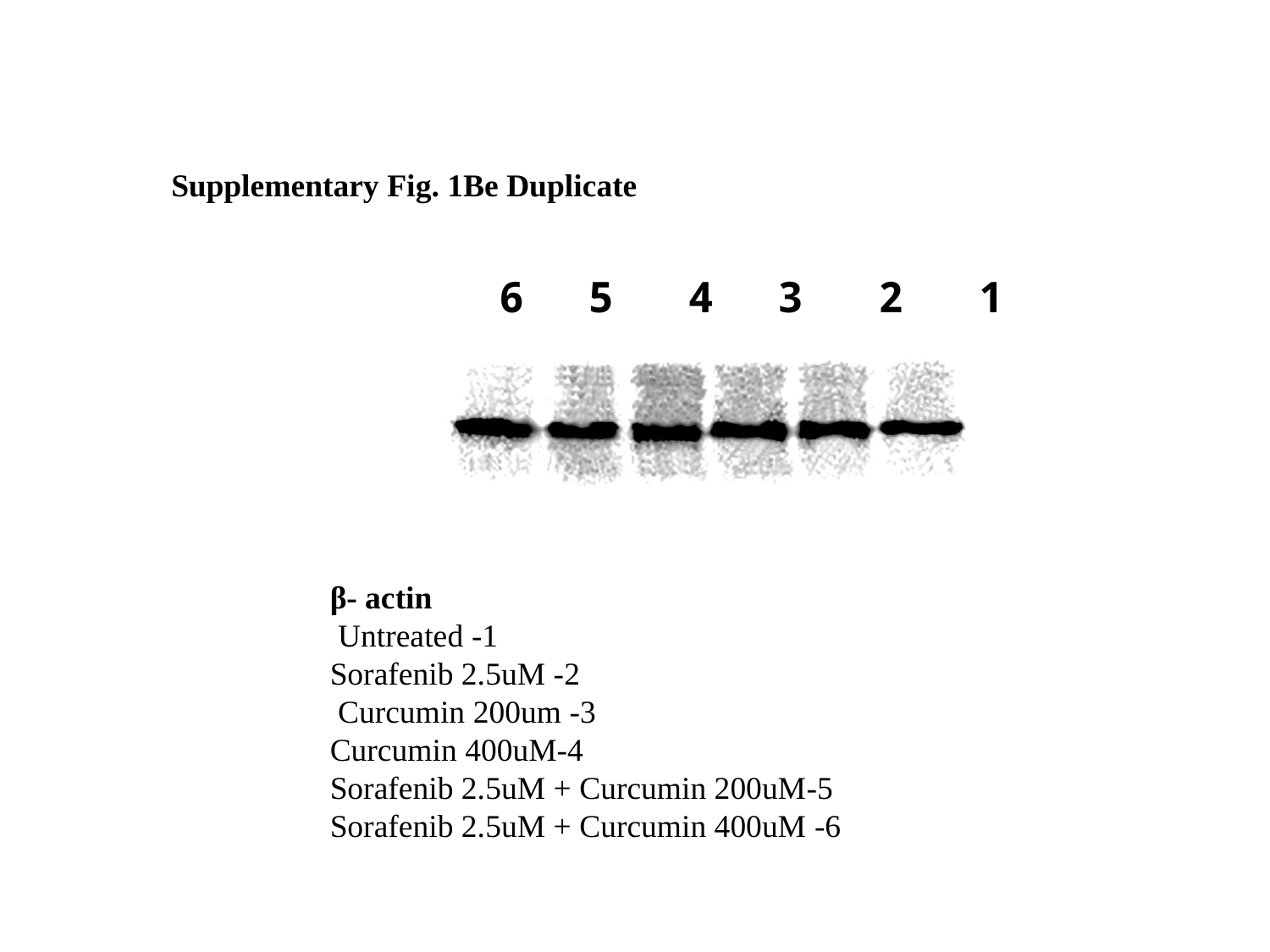

Supplementary Fig. 1Be Duplicate
1 2 3 4 5 6
β- actin
1- Untreated
2- Sorafenib 2.5uM
3- Curcumin 200um
4-Curcumin 400uM
5-Sorafenib 2.5uM + Curcumin 200uM
6- Sorafenib 2.5uM + Curcumin 400uM

## Slide 12
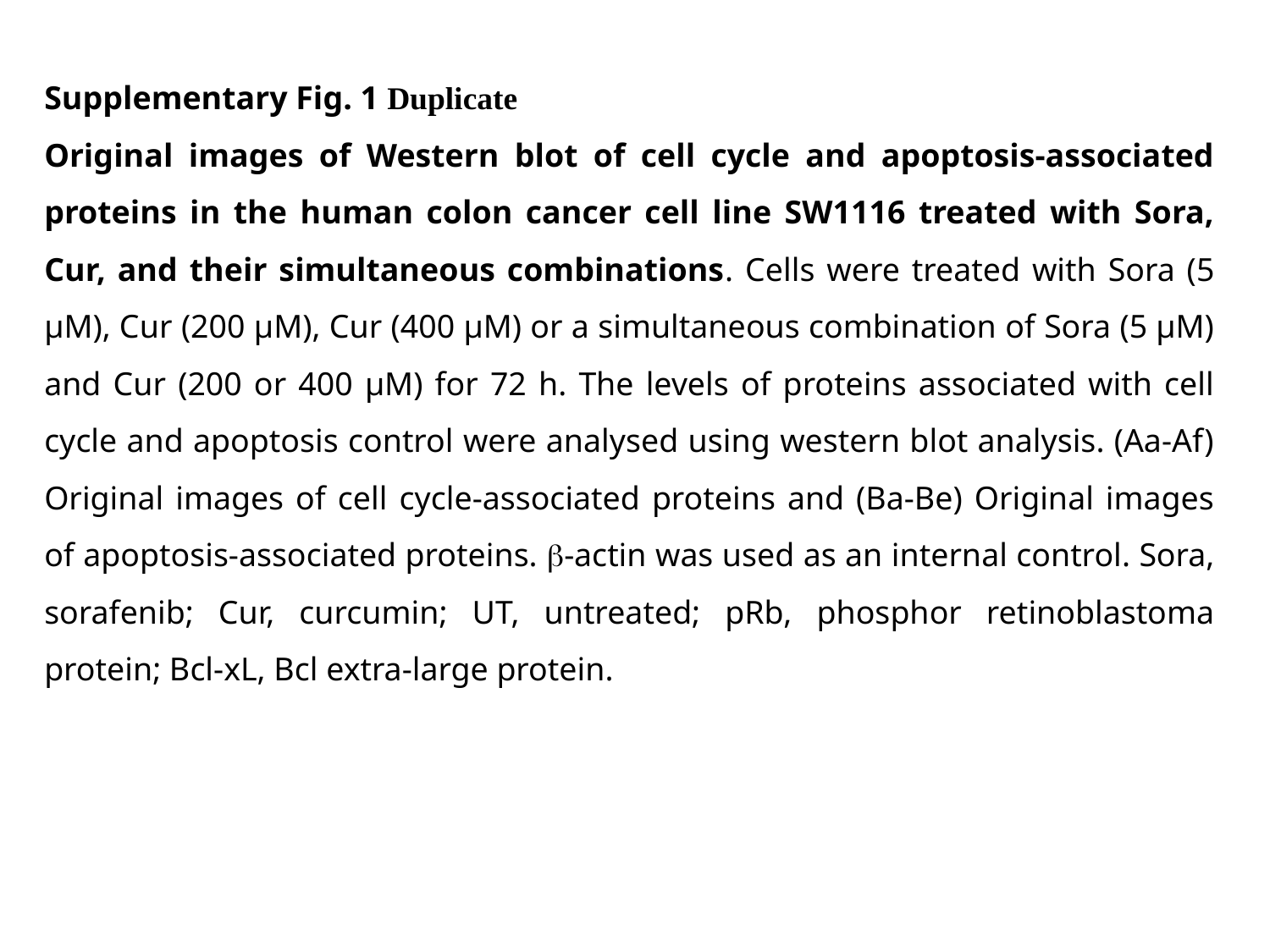

Supplementary Fig. 1 Duplicate
Original images of Western blot of cell cycle and apoptosis-associated proteins in the human colon cancer cell line SW1116 treated with Sora, Cur, and their simultaneous combinations. Cells were treated with Sora (5 µM), Cur (200 µM), Cur (400 µM) or a simultaneous combination of Sora (5 µM) and Cur (200 or 400 µM) for 72 h. The levels of proteins associated with cell cycle and apoptosis control were analysed using western blot analysis. (Aa-Af) Original images of cell cycle-associated proteins and (Ba-Be) Original images of apoptosis-associated proteins. β-actin was used as an internal control. Sora, sorafenib; Cur, curcumin; UT, untreated; pRb, phosphor retinoblastoma protein; Bcl-xL, Bcl extra-large protein.
