# Supplementary material for: Therapeutic efficacy of sorafenib and plant-derived phytochemicals in human colorectal cancer cells
Source: BMC Complement Med Ther. 2023 Jun 26;23:210. doi: 10.1186/s12906-023-04032-6 (PMC10294390; doi:10.1186/s12906-023-04032-6)
Supplement: Supplementary file 1 — Additional file 1. [file 12906_2023_4032_MOESM1_ESM.zip › Fig 1_Suppl_Amended_ 28-5-23.pptx]

## Slide 1
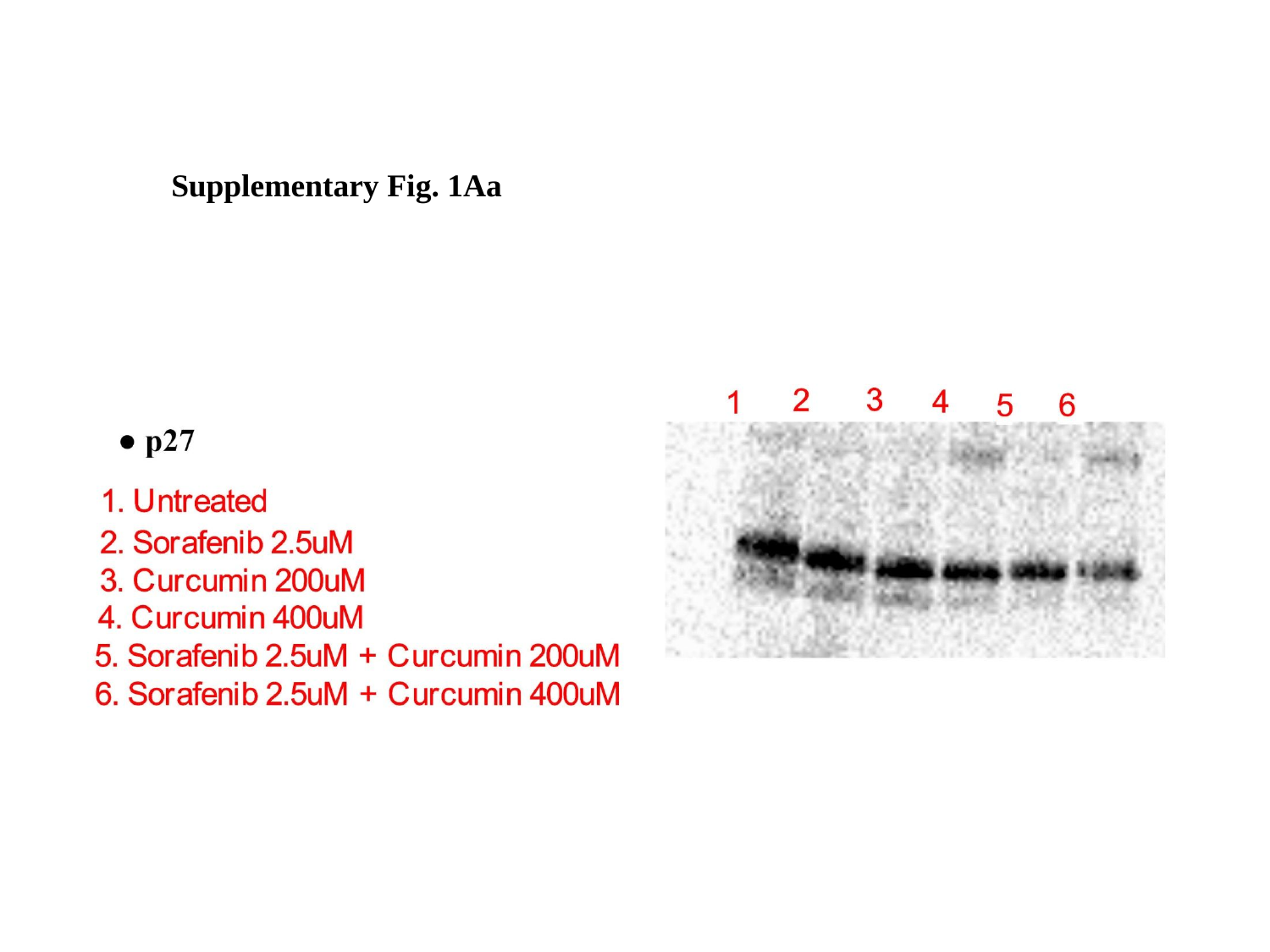

Supplementary Fig. 1Aa

## Slide 2
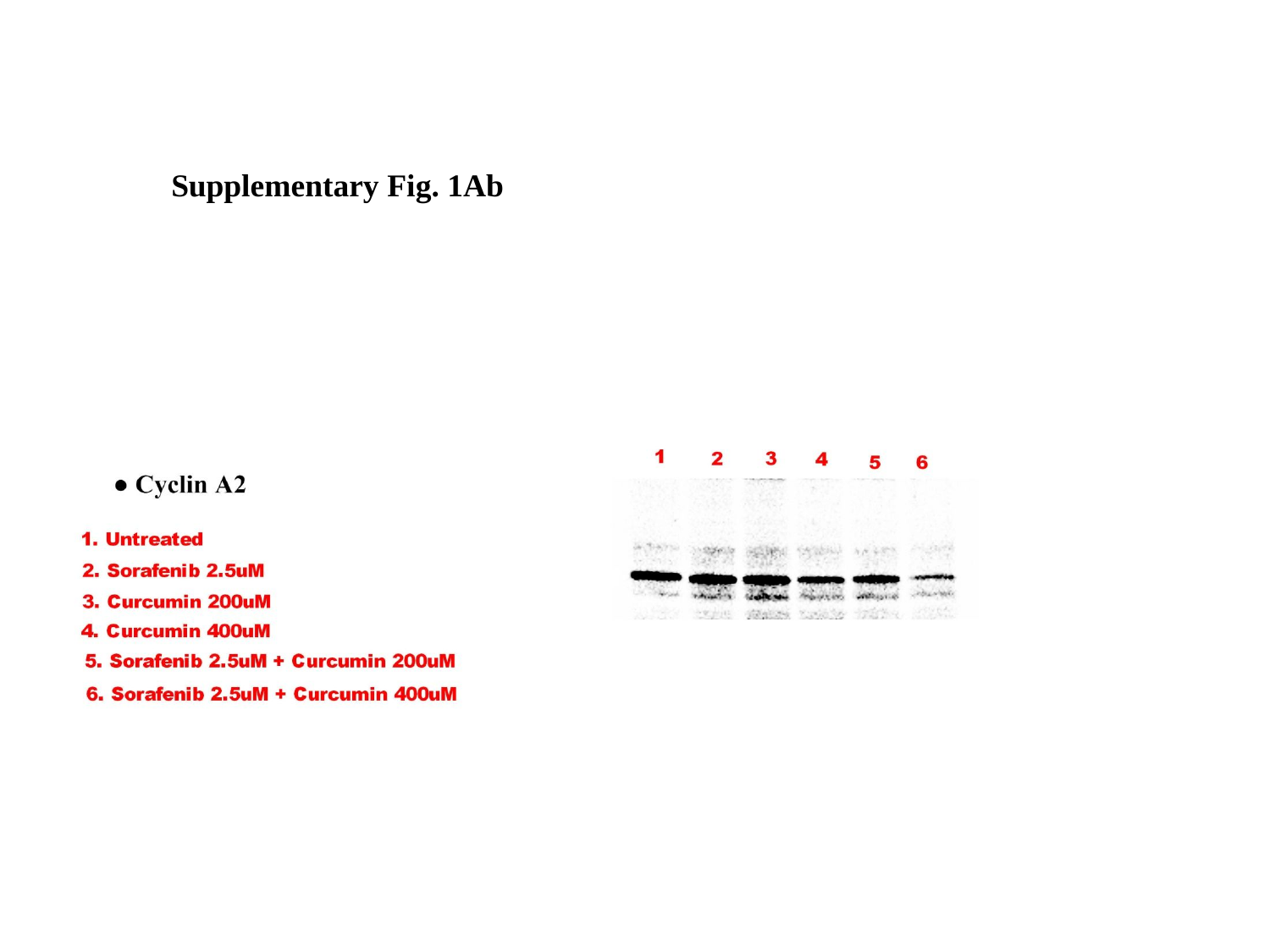

Supplementary Fig. 1Ab

## Slide 3
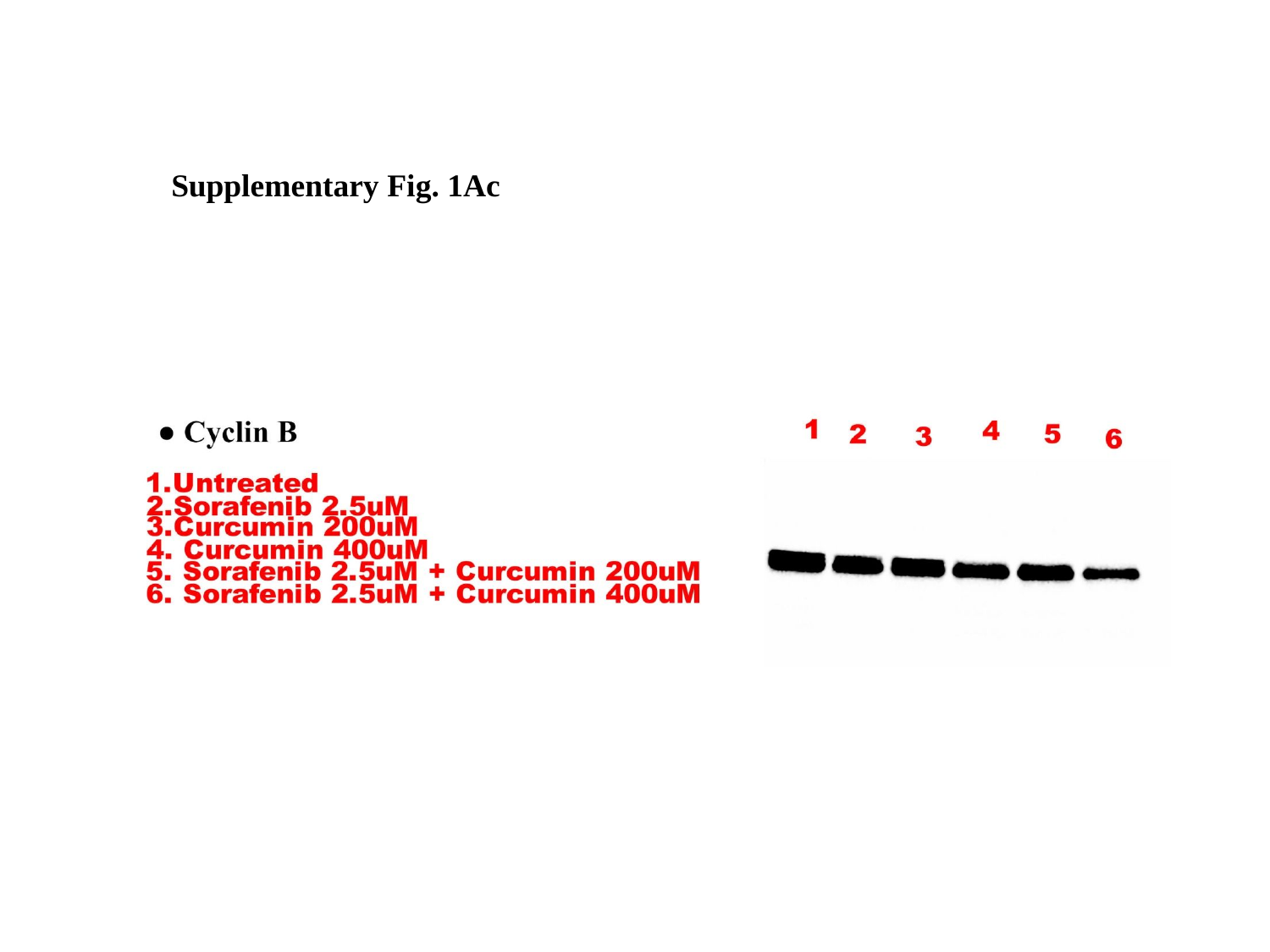

Supplementary Fig. 1Ac

## Slide 4
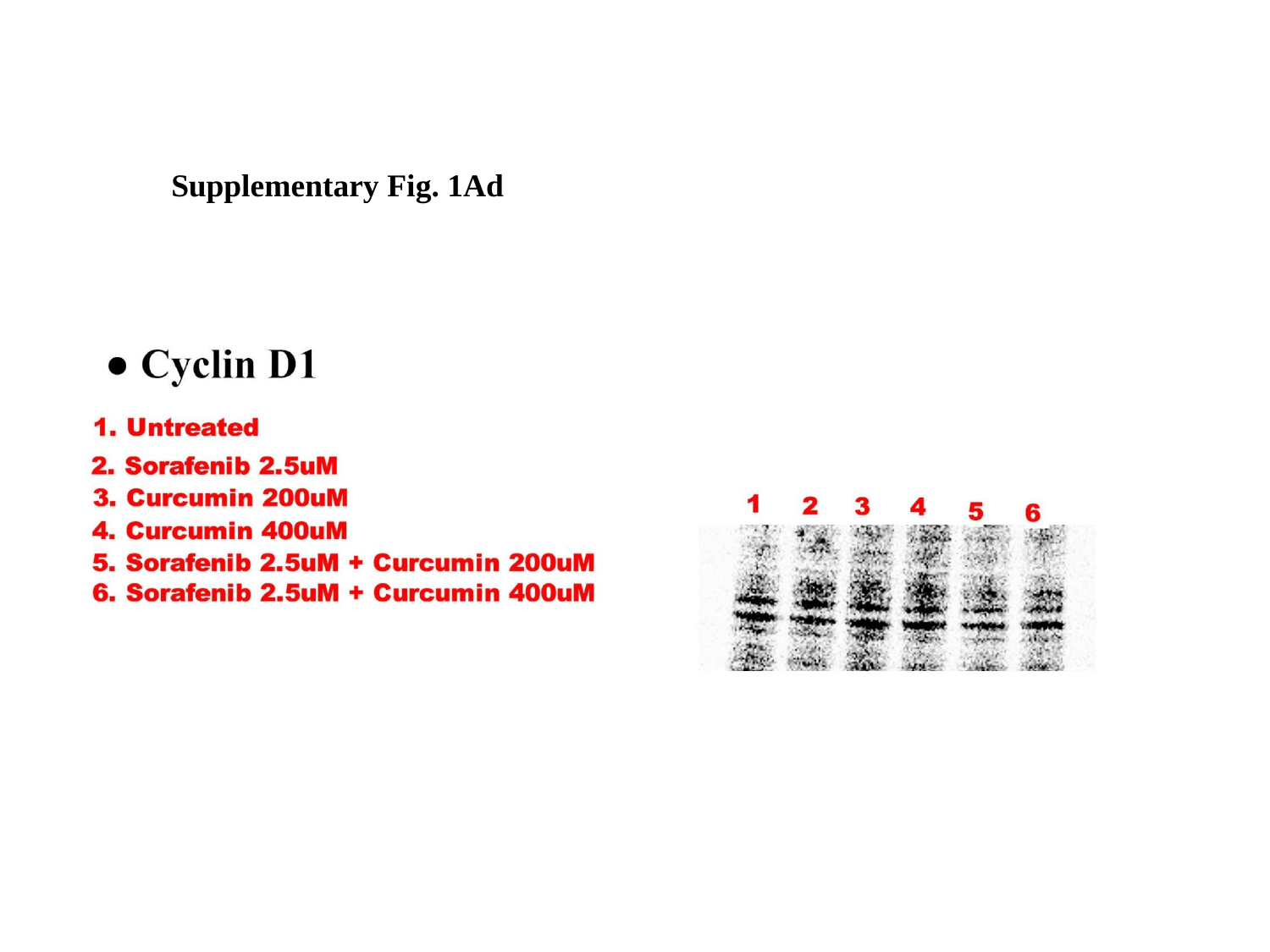

Supplementary Fig. 1Ad

## Slide 5
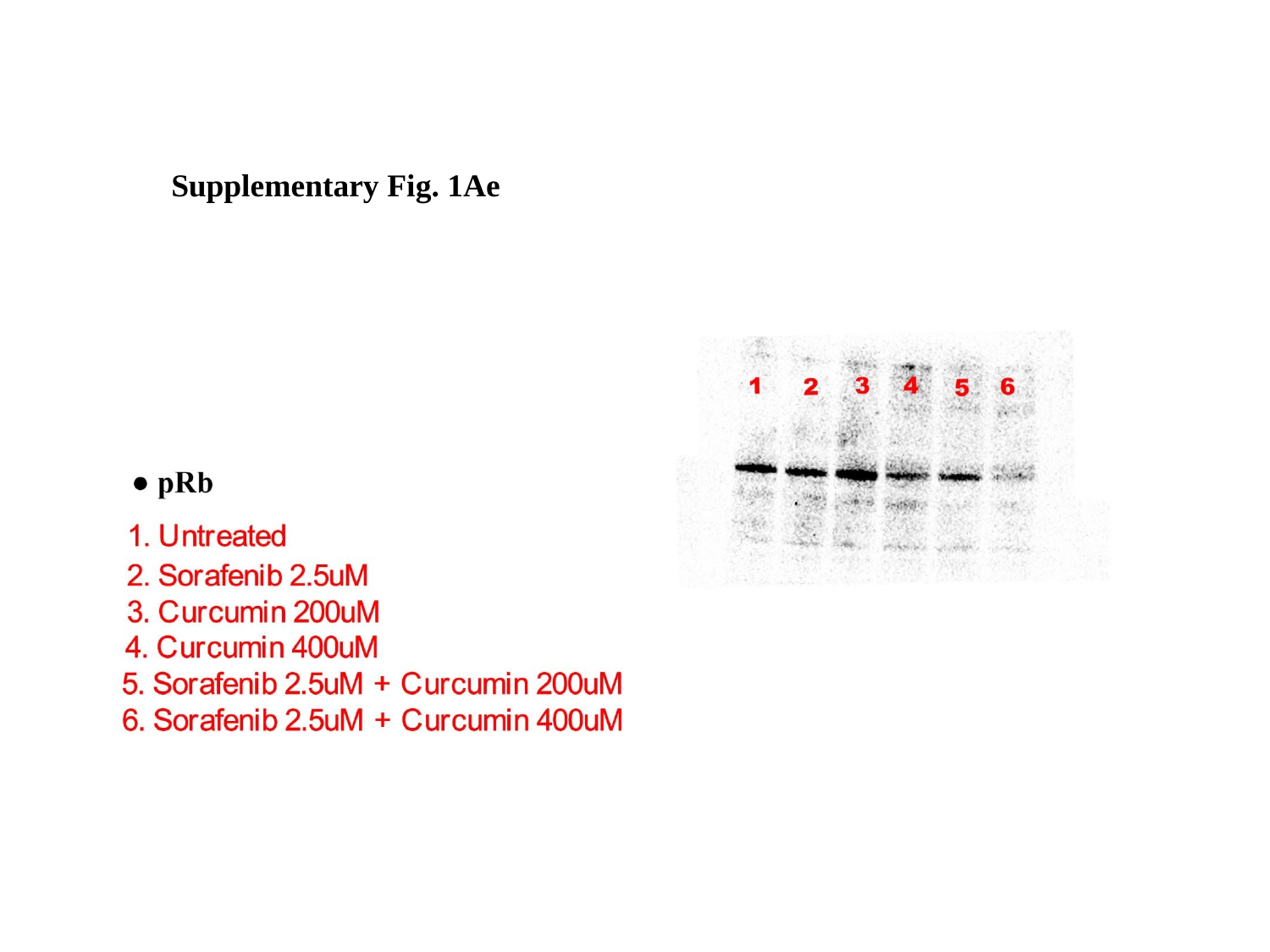

Supplementary Fig. 1Ae

## Slide 6
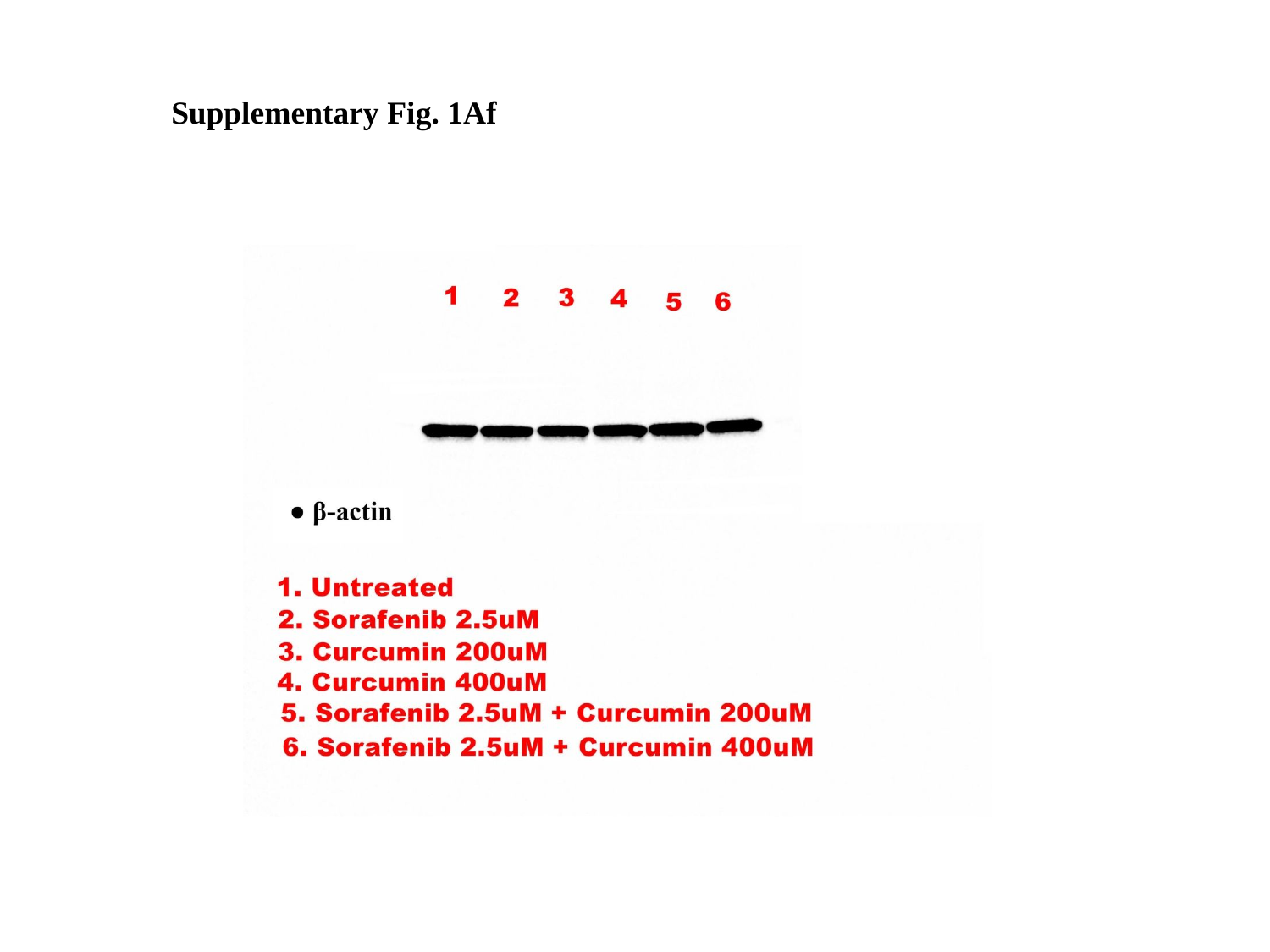

Supplementary Fig. 1Af

## Slide 7
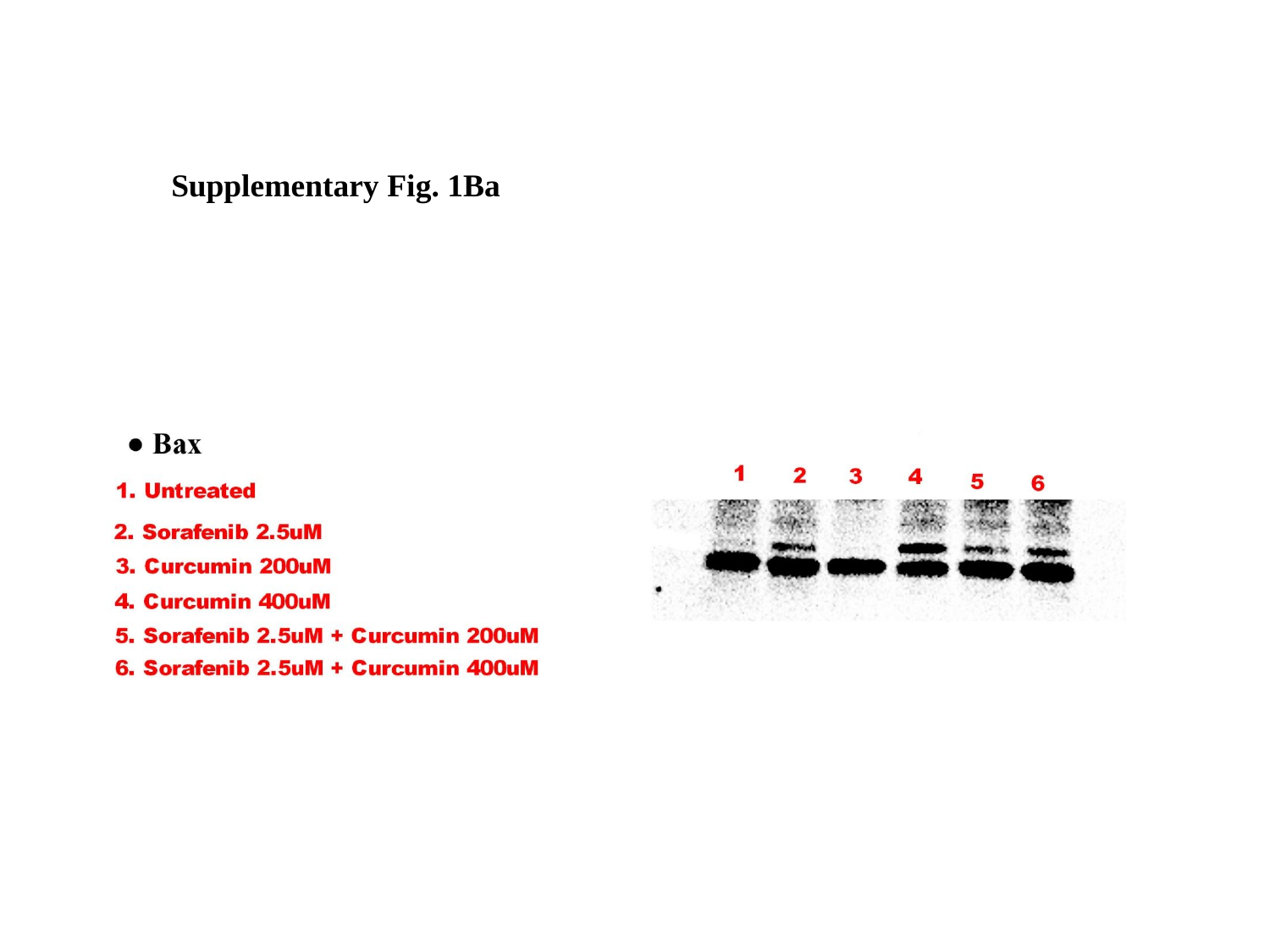

Supplementary Fig. 1Ba

## Slide 8
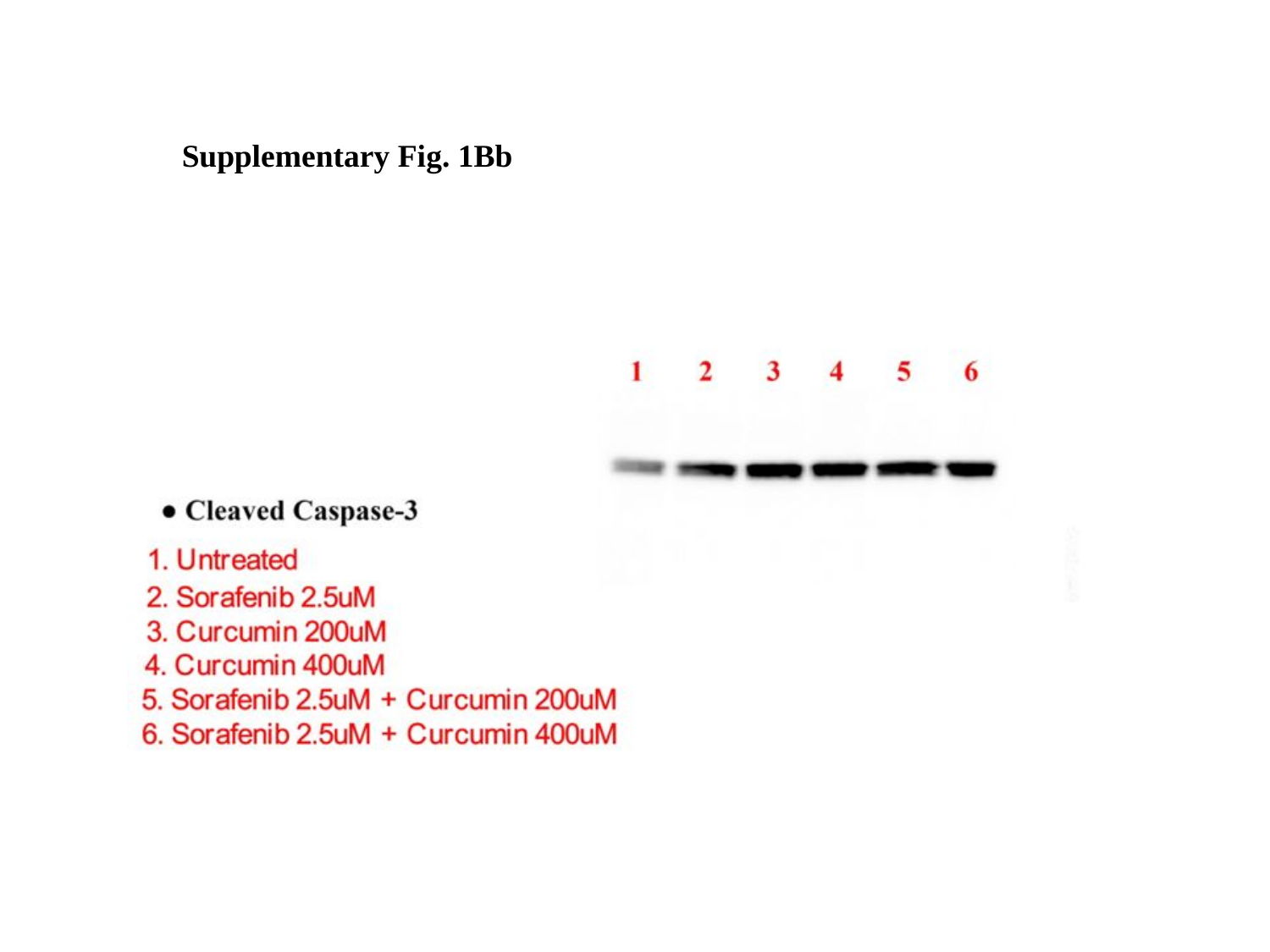

Supplementary Fig. 1Bb

## Slide 9
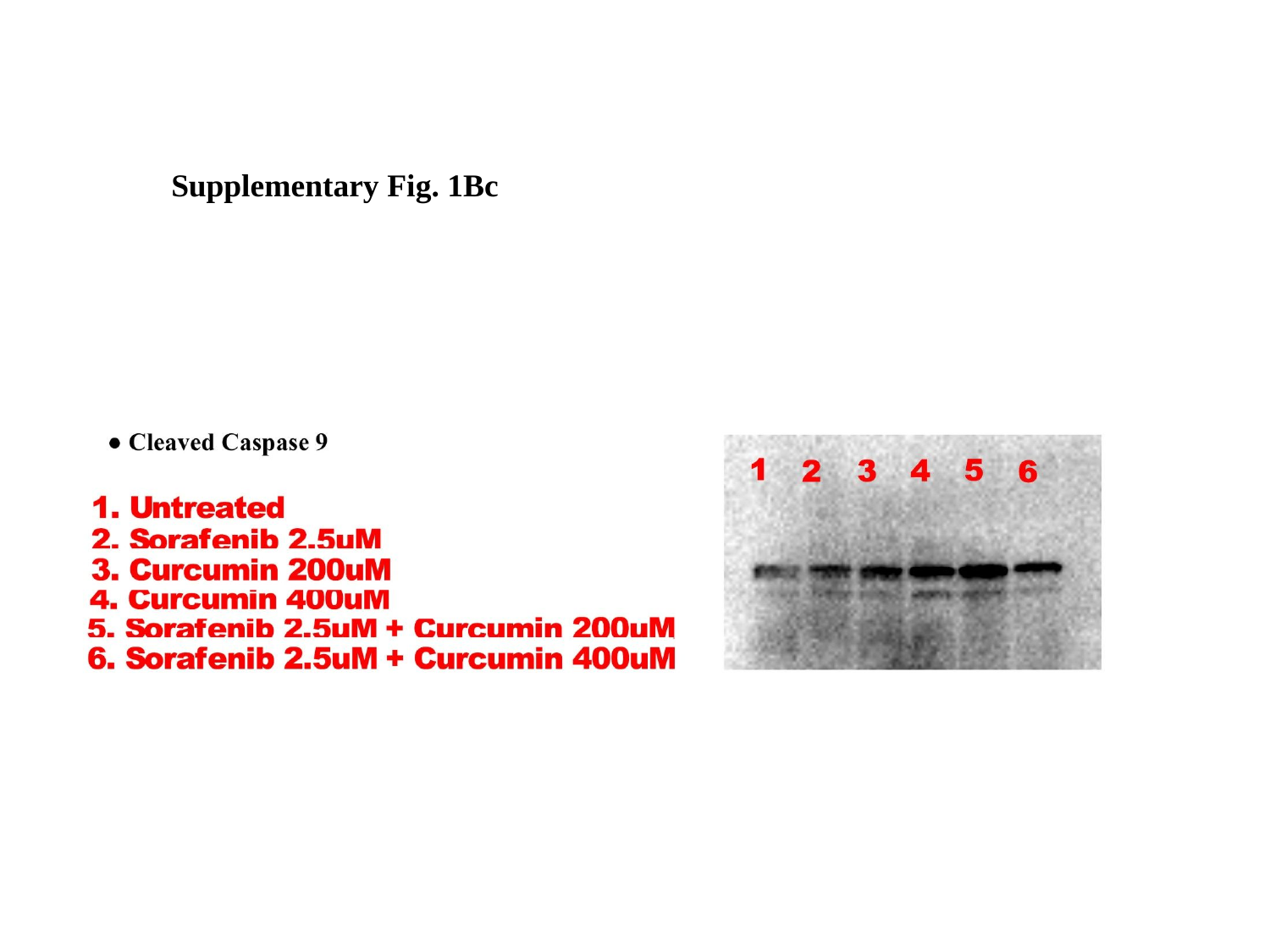

Supplementary Fig. 1Bc

## Slide 10
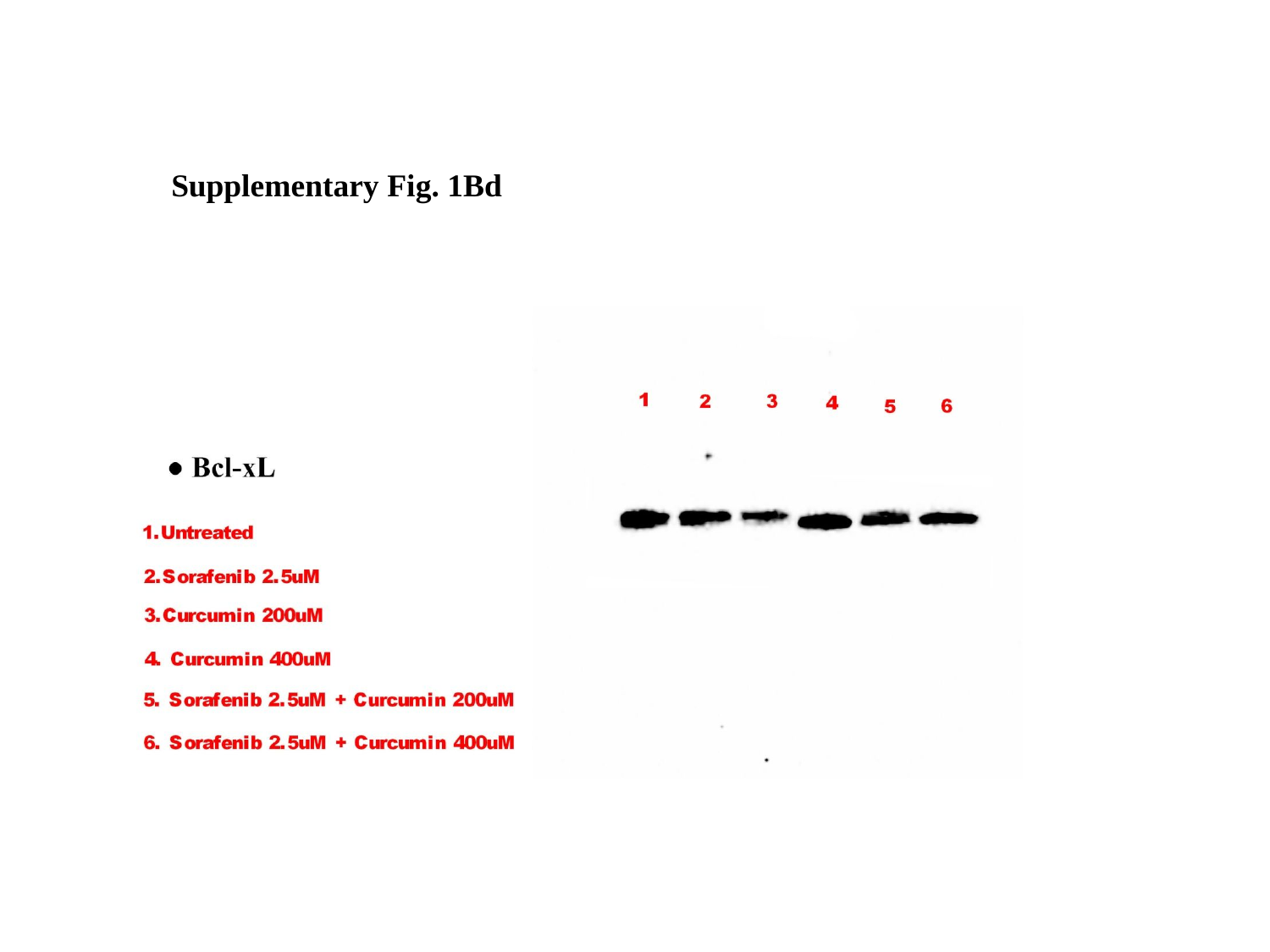

Supplementary Fig. 1Bd

## Slide 11
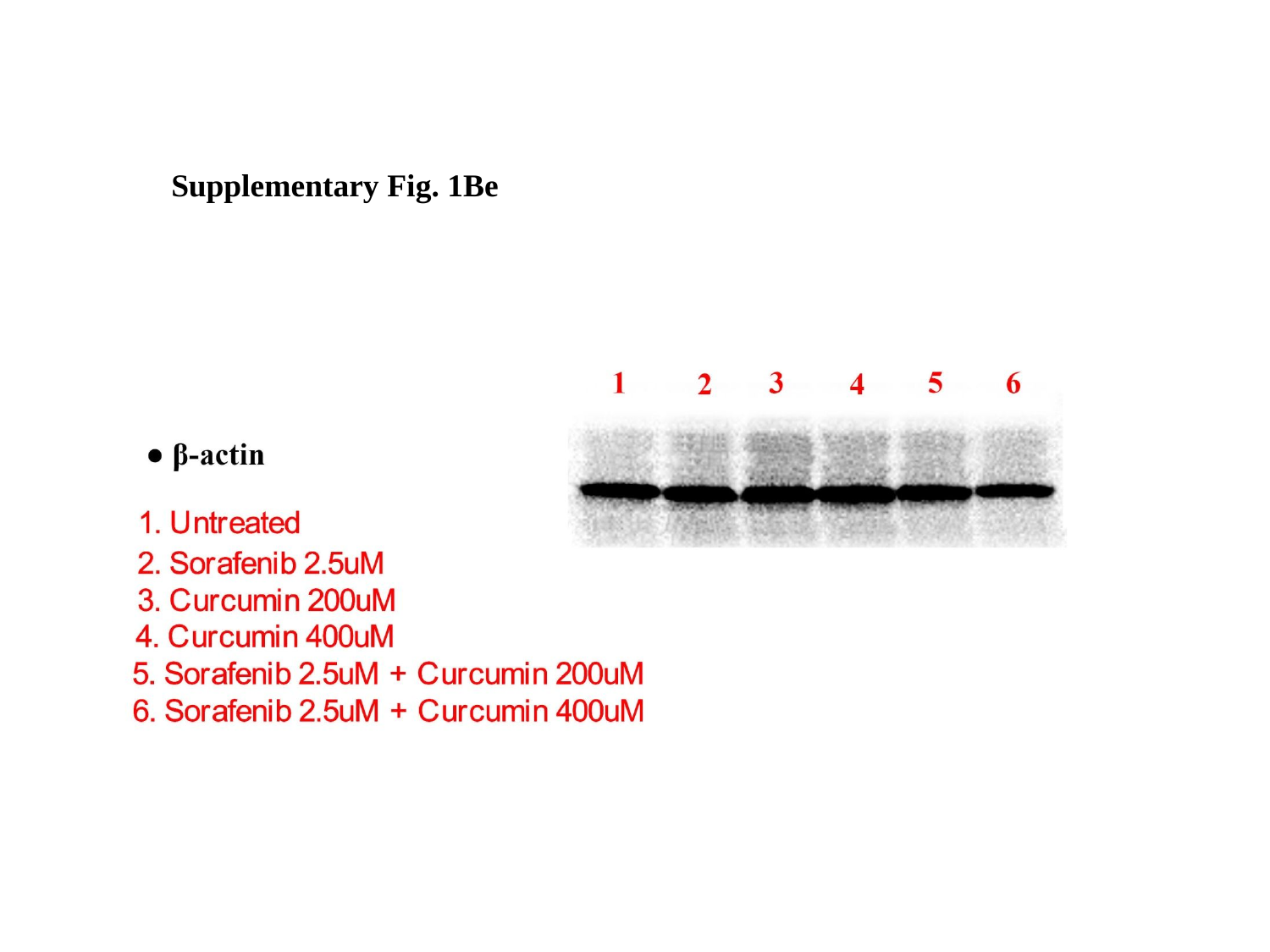

Supplementary Fig. 1Be

## Slide 12
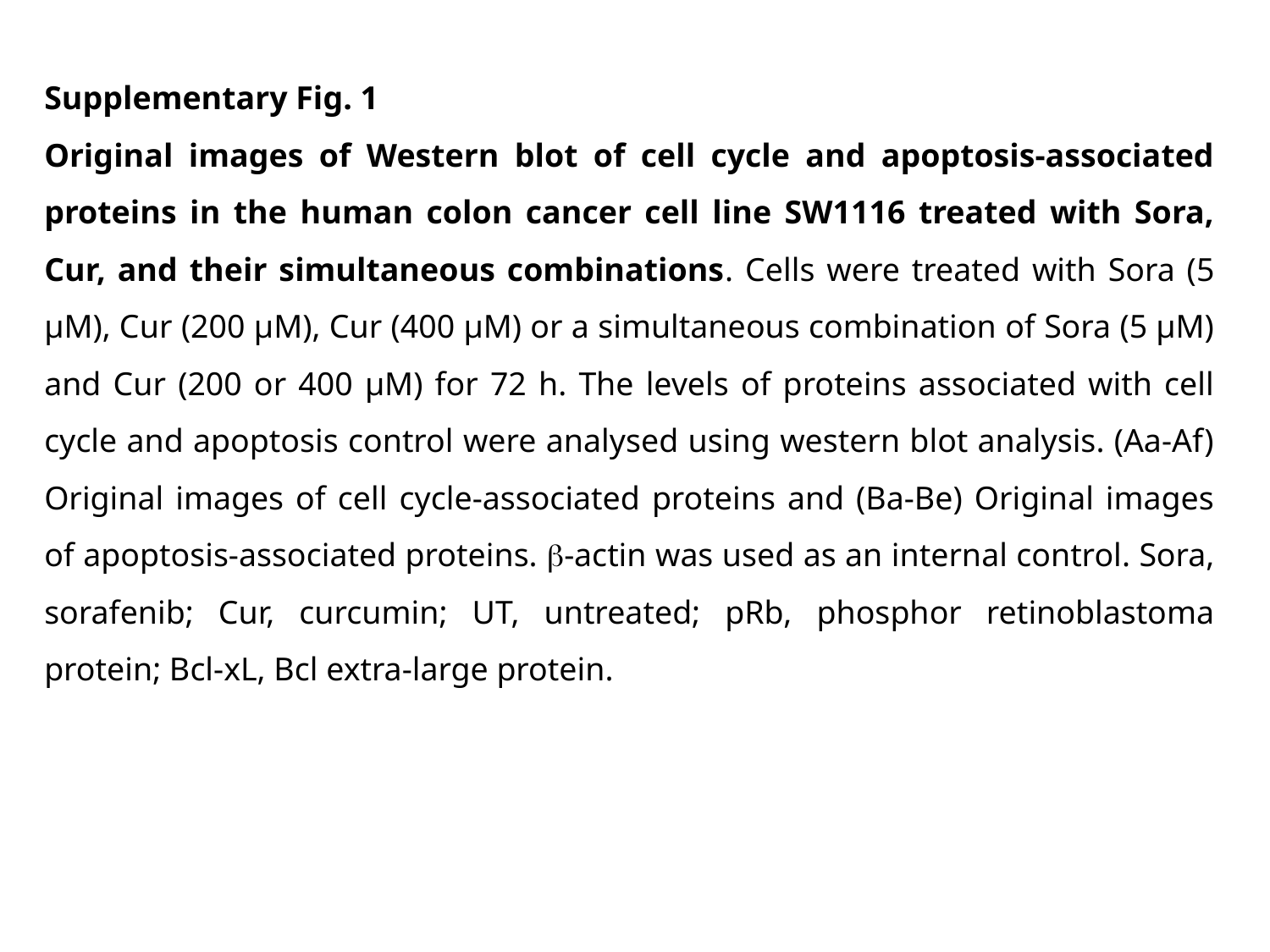

Supplementary Fig. 1
Original images of Western blot of cell cycle and apoptosis-associated proteins in the human colon cancer cell line SW1116 treated with Sora, Cur, and their simultaneous combinations. Cells were treated with Sora (5 µM), Cur (200 µM), Cur (400 µM) or a simultaneous combination of Sora (5 µM) and Cur (200 or 400 µM) for 72 h. The levels of proteins associated with cell cycle and apoptosis control were analysed using western blot analysis. (Aa-Af) Original images of cell cycle-associated proteins and (Ba-Be) Original images of apoptosis-associated proteins. β-actin was used as an internal control. Sora, sorafenib; Cur, curcumin; UT, untreated; pRb, phosphor retinoblastoma protein; Bcl-xL, Bcl extra-large protein.
